# PRMT5 epigenetically regulates the E3 ubiquitin ligase ITCH to influence lipid accumulation during mycobacterial infection

**DOI:** 10.1371/journal.ppat.1010095

**Published:** 2022-06-03

**Authors:** Salik Miskat Borbora, Raju S. Rajmani, Kithiganahalli Narayanaswamy Balaji

**Affiliations:** 1 Department of Microbiology and Cell Biology, Indian Institute of Science, Bangalore, Karnataka, India; 2 Center for Infectious Disease Research, Indian Institute of Science, Bangalore, Karnataka, India; University of Massachusetts Medical School, UNITED STATES

## Abstract

*Mycobacterium tuberculosis* (Mtb), the causative agent of tuberculosis (TB), triggers enhanced accumulation of lipids to generate foamy macrophages (FMs). This process has been often attributed to the surge in the expression of lipid influx genes with a concomitant decrease in those involved in lipid efflux. Here, we define an Mtb-orchestrated modulation of the ubiquitination of lipid accumulation markers to enhance lipid accretion during infection. We find that Mtb infection represses the expression of the E3 ubiquitin ligase, ITCH, resulting in the sustenance of key lipid accrual molecules viz. ADRP and CD36, that are otherwise targeted by ITCH for proteasomal degradation. In line, overexpressing ITCH in Mtb-infected cells was found to suppress Mtb-induced lipid accumulation. Molecular analyses including loss-of-function and ChIP assays demonstrated a role for the concerted action of the transcription factor YY1 and the arginine methyl transferase PRMT5 in restricting the expression of *Itch* gene by conferring repressive symmetrical H4R3me2 marks on its promoter. Consequently, siRNA-mediated depletion of YY1 or PRMT5 rescued ITCH expression, thereby compromising the levels of Mtb-induced ADRP and CD36 and limiting FM formation during infection. Accumulation of lipids within the host has been implicated as a pro-mycobacterial process that aids in pathogen persistence and dormancy. In line, we found that perturbation of PRMT5 enzyme activity resulted in compromised lipid levels and reduced mycobacterial survival in mouse peritoneal macrophages (*ex vivo)* and in a therapeutic mouse model of TB infection (*in vivo)*. These findings provide new insights into the role of PRMT5 and YY1 in augmenting mycobacterial pathogenesis. Thus, we posit that our observations could help design novel adjunct therapies and combinatorial drug regimen for effective anti-TB strategies.

## Author summary

*Mycobacterium tuberculosis* infection leads to the formation of lipid-laden cells (foamy macrophages-FMs) that offer a favorable shelter for its persistence. During infection, we observe a significant reduction in the expression of the E3 ubiquitin ligase, ITCH. This repression allows the sustenance of key lipid accretion molecules (ADRP and CD36), by curbing their proteasomal degradation. Further, we show the repression of ITCH to be dependent on the concerted action of the bifunctional transcription factor, YY1 and the arginine methyl transferase, PRMT5. NOTCH signaling pathway was identified as a master-regulator of YY1 expression. *In vitro* and *in vivo* analyses revealed the significance of PRMT5 in regulating FM formation and consequently mycobacterial burden.

## Introduction

*Mycobacterium tuberculosis* (Mtb), the principal etiological agent of the pulmonary ailment, tuberculosis (TB), continues to co-evolve with the human population making itself one of the major infectious diseases afflicting mankind. Globally in 2020, there were an estimated 1.5 million deaths due to TB, a steep increase, following the onset of the COVID-19 pandemic (WHO, Annual TB Report, 2021).

Upon infecting the host, Mtb coordinates the formation of a highly organized immune structure- the granuloma [1]. TB granulomas are constituted by macrophages, neutrophils, monocytes, dendritic cells, B- and T-cells, fibroblasts, and epithelial cells [2, 3]. Anatomical dissection into the distinct features of the macrophages within the TB granuloma has revealed accumulated lipids as one of the leading physiological countenances. Also referred to as foamy macrophages (FM), these cells act as a protective niche wherein the bacterium thrives and persists. Mtb deregulates the expression of host molecules to trigger increased lipid intake into the cells with a concomitant decrease in the efflux of lipids- thereby contributing to a lipid-rich environment [4]. While Mtb-mediated regulation of key host molecules that contribute to FM formation has been studied at the transcript level; there are scant reports on their post-translational regulation. Parallel studies have highlighted deregulated autophagy [5] and lipophagy [6] to be instrumental in regulating lipid turnover during Mtb infection. To our interest, ubiquitin-dependent degradation of specific proteins implicated in FM formation forms another mechanism which control the stability of proteins that aid in lipid accumulation [7]. In this context, Nedd4-family ligases (a sub-family of HECT-domain containing E3 ligases), among others, are known to play distinctive roles in maintaining the stability of diverse proteins involved in FM formation. Notably, NEDD4-1 can interact with the ATP-Binding Cassette (ABC) transporters- ABCG1 and ABCG4, two essential regulators of cellular lipid efflux [8]. Besides, another report alluded to the role of AIP4 or ITCH in maintaining the levels of the important FM protein, ADRP [9].

During TB, accumulated host lipids have been associated with mycobacterial survival as they provide essential nutrients, contribute to reduced antigen presentation [10, 11, 12] and aid in pathogen latency and reactivation [13]. A separate study revealed that knocking down *Nedd4* in THP-1 macrophages elicited higher CFU of Mtb and other intracellular pathogens [14]. This encouraged us to investigate NEDD4-family of E3 ligases in the context of Mtb-driven FM formation and consequently pathogen survival.

Pathogen-driven epigenetic changes have been indicated to govern the expression of distinct molecules that contribute to mycobacterial survival [4, 15, 16, 17, 18]. Emerging literature have indicated towards epigenetic regulation of NEDD4-family ligases, where HDAC2-dependent downregulation of histone methyltransferase Ehmt2 (G9a) was shown to activate NEDD4[19]. While a plethora of epigenetic regulators have been associated with the progression of several infectious diseases, including TB [4, 16, 18, 20, 21], there is a dearth of reports on the role of arginine methyl transferases such as Protein arginine methyl transferases (PRMTs) during distinct infections and the specific regulation of E3 ligases. Of all the characterized PRMTs, PRMT1, PRMT2, PRMT3, PRMT6, PRMT8 and CARM1 catalyze asymmetric methylation, while PRMT5 and PRMT9 bring about symmetric methylation on specific arginine residues [22]. Available reports have demonstrated the role of PRMT5 in regulating lipid biogenesis. Notably, PRMT5 has been shown to arbitrate seipin-mediated lipid droplet biogenesis in adipocytes [23]. Besides, PRMT5 could enhance lipogenesis in cancer cells by methylating SREBP1a [24]. These reports encouraged us to investigate a PRMT5-mediated regulation of FM formation during Mtb infection.

In this study, we sought to understand the role of Mtb-mediated post-translational regulation of key molecules in the formation of FMs. Employing the use of siRNA and pharmacological inhibitors, we highlight the role of PRMT5 in repressing the expression of the E3 ligase, ITCH, which contributed to lipid accumulation during Mtb infection both *in vitro* and *in vivo*.

## Results

### Mycobacterial infection suppresses the expression of the E3 ubiquitin ligase, ITCH, to enhance lipid accumulation in cells

**Fig 1:**
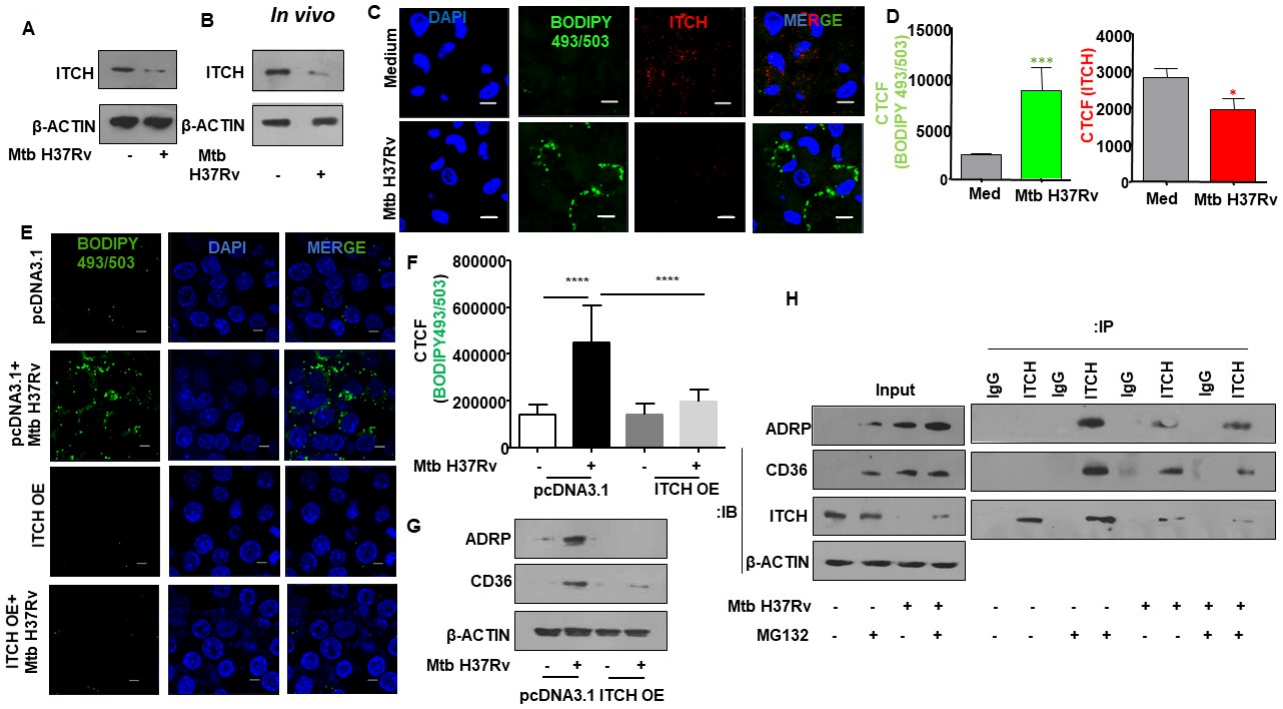
E3 ubiquitin ligase ITCH (AIP4) repression aids in Mtb-mediated enhanced lipid accumulation. **(A)** Mouse peritoneal macrophages were infected with Mtb H37Rv for 24 h. Whole cell lysates were assessed for ITCH protein expression by immunoblotting. **(B)** BALB/c mice were aerosol-infected with Mtb H37Rv for 28 days. Protein levels of ITCH was analyzed in the lung homogenates of uninfected and infected mice by immunoblotting. **(C, D)** Mouse peritoneal macrophages were infected with Mtb H37Rv for 48 h and assessed for lipid accumulation (BODIPY493/503) and ITCH expression by immunofluorescence microscopy; **(C)** representative image and **(D)** respective quantification. **(E, F)** RAW 264.7 cells were transfected with pcDNA3.1 and ITCH OE vectors. Transfected cells were infected with Mtb H37Rv for 48 h and assessed for the accumulation of lipids by fluorescence microscopy (BODIPY493/503); **(E)** representative image and **(F)** respective quantification. **(G)** RAW 264.7 cells were transfected with pcDNA3.1 and ITCH OE vectors; Transfected cells were infected with Mtb H37Rv for 24 h and whole cell lysates were assessed for the levels of the indicated lipid accumulation markers by immunoblotting. **(H)** Mouse peritoneal macrophages were infected with Mtb H37Rv for 24 h and treated with MG132 for the last 4 h of infection. Whole cell lysates were immunoprecipitated with IgG control or ITCH antibodies and assessed for their interaction with the indicated lipid accumulation markers by immunoblotting. All immunoblotting and immunofluorescence data are representative of three independent experiments. Lung homogenates from at least three mice were independently assessed for ITCH expression by immunoblotting. β-ACTIN was utilized as loading control. Med, medium; OE, over expression; CTCF, corrected total cell fluorescence. *, p < 0.05; *** p < 0.001; ****, p < 0.0001 (Student’s t-test in D, One way ANOVA in F; GraphPad Prism 6.0). Scale bar, 5 μm.

Lipid droplets are formed by the coordinated action of diverse processes which include its biogenesis, maturation, and turnover [25]. As introduced, ubiquitination has been implicated in the regulation of proteins that contribute to lipid turnover within cells [26], thereby providing distinct regulatory potential to the entire process of lipid accumulation [27]. Since Nedd4-family ligases were reported to be responsible for ubiquitination and degradation of lipid-associated proteins such as ADRP [9], we screened for Nedd4-family ligases that are differentially expressed during Mtb infection. Interestingly, we found the transcript levels of the E3 ligase *Itch* to be significantly downregulated upon mycobacterial infection both *in vitro* (S1A Fig) and *in vivo* (S1B Fig), prompting us to investigate the molecule further. This was corroborated at the protein level as Mtb-infected mouse peritoneal macrophages displayed diminished levels of ITCH protein (Fig 1A), while homogenates from the lungs of Mtb-infected mice exhibited a similar downregulated expression of ITCH (Fig 1B). Furthermore, immunofluorescence analysis revealed enhanced accumulation of lipids with a concomitant decrease in the levels of ITCH expression upon Mtb infection in murine peritoneal macrophages (Fig 1C and 1D). Also, transient over expression of ITCH in RAW 264.7 macrophages compromised the Mtb-induced FM formation, thereby emphasizing on the role of ITCH in lipid accumulation (Fig 1E and 1F).

Previously, the role of ADRP and CD36 in Mtb-triggered FM formation was underscored as siRNA-mediated knock down of ADRP and/ or CD36 resulted in diminished lipid pools within Mtb-infected macrophages [4]. Over expression of ITCH compromised the elevated levels of ADRP and CD36 (Fig 1G). This led us to assess the possible role of ITCH in the proteasomal degradation of the key lipid accumulation molecules. We found a significant degree of interaction of ITCH with ADRP and CD36 in the uninfected scenario (Fig 1H), suggesting the contribution of ITCH in maintaining lipid homeostasis in macrophages under basal conditions. However, in the event of ITCH repression during Mtb infection, ADRP and CD36 levels are sustained, thereby aiding in lipid accumulation. Together, these observations indicate a role for ITCH in regulating FM formation during Mtb infection.

### Transcription regulator, YY1, aids in Mtb-mediated repression of ITCH

**Fig 2:**
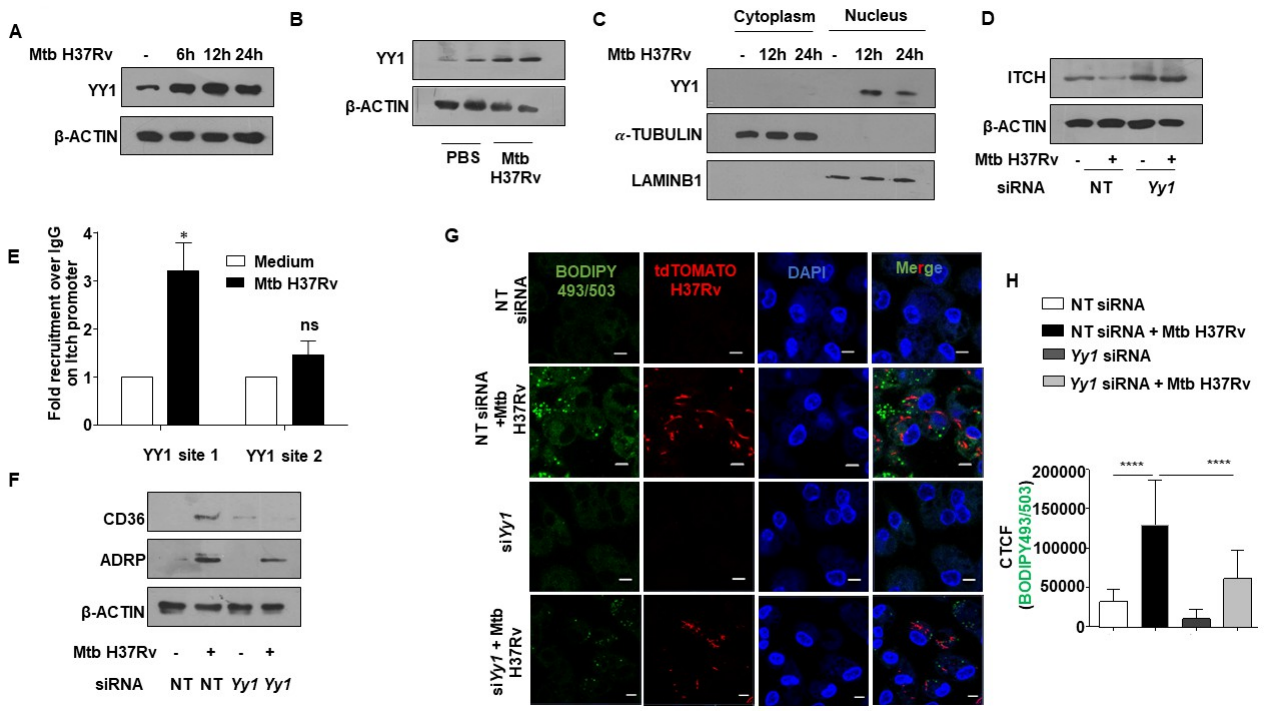
Transcription factor YY1 regulates ITCH repression during Mtb infection. **(A)** Mouse peritoneal macrophages were infected with Mtb H37Rv for the indicated time points and YY1 expression was analyzed in the whole cell lysates by immunoblotting. **(B)** BALB/c mice were aerosol-infected with Mtb H37Rv for 28 days. Protein levels of YY1 was analyzed in the lung homogenates of uninfected and infected mice by western blotting. **(C)** Mouse peritoneal macrophages were infected with Mtb H37Rv for the indicated time points. Cells were partitioned into nuclear and cytoplasmic fractions and assessed for the nuclear translocation of YY1 by immunoblotting. **(D)** Mouse peritoneal macrophages were transfected with NT or *Yy1* siRNAs. Transfected cells were infected with Mtb H37Rv for 24 h and assessed for the expression of ITCH by immunoblotting. **(E)** Mouse peritoneal macrophages were infected with Mtb H37Rv for 24 h and assessed for the recruitment of YY1 over the *Itch* promoter by ChIP assay. **(F)** Mouse peritoneal macrophages were transfected with NT or *Yy1* siRNAs. Transfected cells were infected with Mtb H37Rv for 24 h and assessed for the expression of ADRP and CD36 by immunoblotting. **(G, H)** Mouse peritoneal macrophages were transfected with NT or *Yy1* siRNAs. Transfected cells were infected with tdTomato Mtb H37Rv for 48 h and analyzed for lipid accumulation (BODIPY493/503) by confocal microscopy; **(G)** representative image and **(H)** respective quantification. All immunoblotting, immunofluorescence and ChIP data are representative of three independent experiments. Lung homogenates from at least three mice were independently assessed for YY1 expression by immunoblotting. β-ACTIN, *α*-TUBULIN and LAMINB1 were utilized as loading control. NT, non-targeting; CTCF, corrected total cell fluorescence; ChIP, Chormatin immunoprecipitation; ns, non-significant. *, p < 0.05; ****, p < 0.0001 (Student’s t-test in E, One way ANOVA in H; GraphPad Prism 6.0). Scale bar, 5 μm.

Next, we analyzed the promoter region of *Itch* gene to identify the potential regulators of ITCH expression. Bioinformatic assessment of the 2kb upstream sequence revealed two putative binding sites of the transcriptional regulator, YY1. A previous report suggests an enhanced expression of YY1 in human TB samples, where it plays a cardinal role in the regulation of specific cytokines [28]. Essentially, YY1 is a zinc finger domain containing protein that can activate or repress genes based on its interacting partners [29]. Mouse peritoneal macrophages infected with Mtb displayed an enhanced expression of YY1 in a time-dependent manner (Fig 2A). A similar trend was observed *in vivo* as elevated YY1 expression was observed in the lung homogenates of Mtb-infected mice (Fig 2B). Analysis of the subcellular localization of YY1 in murine macrophages revealed that Mtb infection enhanced the nuclear localization of YY1 (Fig 2C), underscoring the possible role of YY1 in mediating transcriptional processes upon Mtb infection. Next, we compromised *Yy1* levels in murine macrophages with targeted siRNA and found a marked rescue in the Mtb-driven repression of ITCH (Fig 2D). This was further confirmed by ChIP assay, that indicated an enhanced recruitment of YY1 at its respective binding sites on the promoter of *Itch* (Fig 2E).

With the premise of YY1-dependent downregulation of ITCH, we verified the contribution of YY1 in FM generation during Mtb infection. We found the expression of the FM markers, ADRP and CD36, to be compromised in Mtb-infected macrophages transfected with *Yy1* siRNA (Fig 2F). Furthermore, depletion of *Yy1* in mouse peritoneal macrophages also reduced lipid accumulation in Mtb-infected macrophages as assessed by BODIPY 493/503 staining (Fig 2G and 2H).

### NOTCH signaling pathway contributes to mycobacteria-induced lipid accumulation through YY1

Perturbation of host signaling pathways has been shown to result in FM formation during Mtb infection. Notably, TNF receptor signaling through the downstream activation of the caspase cascade and the mammalian target of rapamycin complex 1 (mTORC1) is central to triglyceride accumulation in human macrophages infected with Mtb [30]. Besides, NOTCH, EGFR, and PI3K pathways have been separately implicated in Mtb-driven FM formation [4, 6].

**Fig 3:**
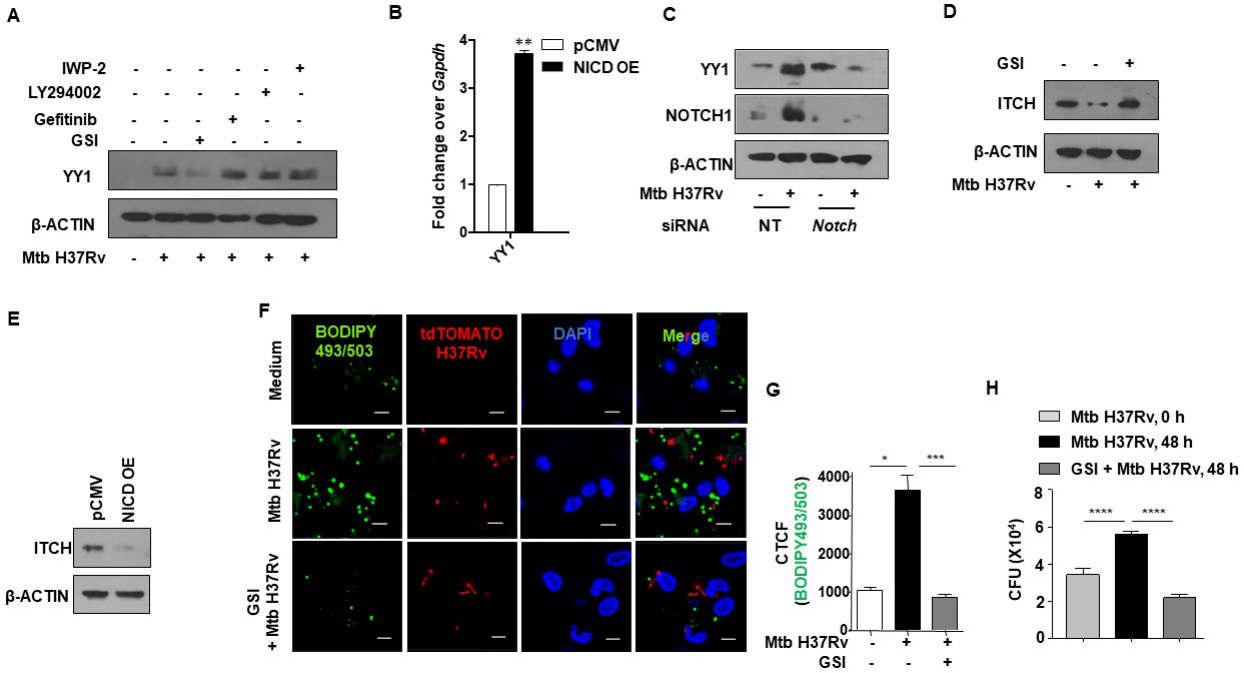
Activated NOTCH signaling pathway aids in enhanced YY1 expression during Mtb infection. **(A)** Mouse peritoneal macrophages were treated with the indicated inhibitors for 1 h, followed by 24 h infection with Mtb H37Rv. Whole cell lysates were assessed for the expression of YY1 by immunoblotting. **(B)** RAW 264.7 cells were transfected with pCMV or NICD overexpression vectors and assessed for the transcript levels of *Yy1* by qRT-PCR. **(C)** Mouse peritoneal macrophages were transfected with NT or *Notch* siRNAs. Transfected cells were infected with Mtb H37Rv for 24 h and assessed for the expression of YY1 and NOTCH1 by immunoblotting. **(D)** Mouse peritoneal macrophages were treated with NOTCH pathway inhibitor GSI for 1 h, followed by 24 h infection with Mtb H37Rv. Whole cell lysates were assessed for the expression of ITCH by immunoblotting. **(E)** RAW 264.7 cells were transfected with pCMV control or NICD overexpression vectors and assessed for the expression of ITCH by immunoblotting. **(F, G)** Mouse peritoneal macrophages were treated with the NOTCH pathway inhibitor GSI for 1 h, followed by 48 h infection with tdTomato Mtb H37Rv and analyzed for lipid accumulation (BODIPY493/503) by confocal microscopy; **(F)** representative image and **(G)** respective quantification. **(H)** Mouse peritoneal macrophages were infected with Mtb H37Rv for 4 h. Extracellular bacteria were removed, and the infected cells were cultured in the presence or absence of PRMT5 inhibitor, EPZ015666 for 48 h. Cells were lysed and plated on 7H11 to enumerate intracellular Mtb H37Rv burden. qRT-PCR data represents mean±S.E.M. and immunoblotting and immunofluorescence data are representative of three independent experiments. OE, over expression; NT, non-targeting; CTCF, corrected total cell fluorescence; CFU, colony forming units. IWP-2, WNT pathway inhibitor; LY294002, PI3K pathway inhibitor; Gefitinib, EGFR pathway inhibitor; GSI, Gamma secretase inhibitor (GSI), NOTCH pathway inhibitor. *, p < 0.05; **, p < 0.01; *** p < 0.001; ****, p < 0.0001 (Student’s t-test in D, One way ANOVA in G,H; GraphPad Prism 6.0). Scale bar, 5 μm.

With this premise, using specific pharmacological interventions against distinct cellular pathways (IWP-2: WNT pathway inhibitor; LY294002: PI3K pathway inhibitor; Gefitinib: EGFR pathway inhibitor; GSI, Gamma secretase inhibitor (GSI): NOTCH pathway inhibitor), we found the possible role of NOTCH signaling in the expression of YY1 as inhibition of the pathway using GSI compromised the ability of Mtb to induce the expression of YY1 in macrophages (Fig 3A). NOTCH pathway activation is characterized by the cleavage of the intracellular domain of the NOTCH receptor (NICD) by the enzyme Gamma secretase. GSI blocks the activity of Gamma secretase and thereby inhibits the activation of the NOTCH pathway. In line with previous reports [31, 32], we found an activation of NOTCH signaling pathway (elevated NICD expression) in mouse peritoneal macrophages infected with Mtb (S3A Fig). Corroborating this observation, the transcription factor, HES1, a bona fide target of NOTCH signaling, was also found to be upregulated upon Mtb infection as assessed by transcript analysis (S3B Fig) and luciferase reporter assay (S3C Fig).

To verify the role of NOTCH pathway activation in augmenting YY1 levels, we overexpressed NICD in RAW 264.7 macrophages and found enhanced levels of YY1 transcript even in the absence of Mtb infection, underscoring the role of NOTCH in the elevated expression of YY1 (Fig 3B). Further, Mtb-mediated expression of YY1 was compromised in macrophages expressing *Notch1* siRNA (Fig 3C). Additionally, perturbation of the NOTCH pathway in murine peritoneal macrophages using GSI alleviated the Mtb-mediated diminished expression of ITCH (Fig 3D); while NICD overexpressing macrophages displayed compromised expression of ITCH (Fig 3E), validating our observations on the role of the NOTCH pathway in regulating the levels of YY1 and consequently the E3 ligase, ITCH. Furthermore, inhibition of the NOTCH pathway hindered the accumulation of lipids during Mtb infection (Fig 3F and 3G) thereby endorsing the significant role of NOTCH-YY1-ITCH axis in Mtb-induced FM formation.

Both NOTCH signaling and lipid accumulation was shown to aid mycobacterial survival within the host [31, 4]. In this context, using *in vitro* CFU analysis, we found that perturbation of NOTCH pathway (using GSI) severely compromised mycobacterial survival (Fig 3H), without any considerable effect on the bacterial uptake (S4A Fig).

### PRMT5 imparts repressive methylation signature on the promoter of ITCH during mycobacterial infection

**Fig 4:**
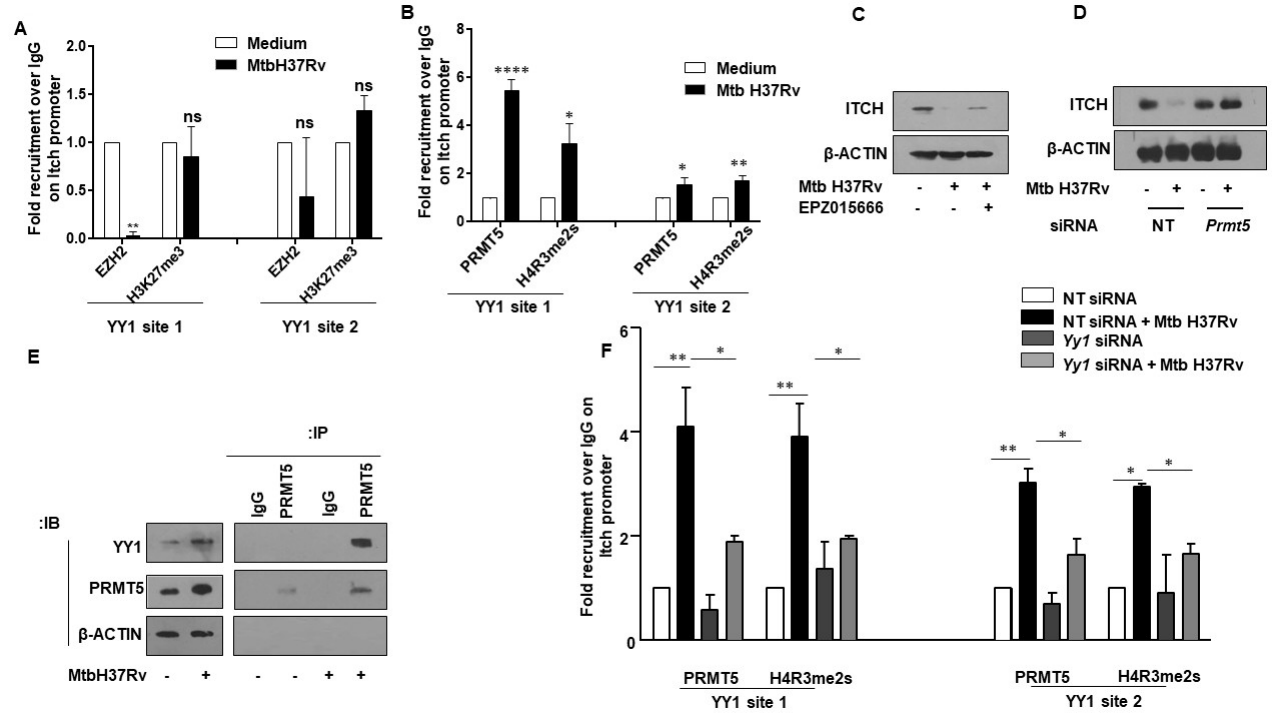
YY1 associates with the arginine methyl transferase PRMT5 to regulate ITCH repression during Mtb infection. **(A)** Mouse peritoneal macrophages were infected with Mtb H37Rv for 24 h and assessed for the recruitment of EZH2 and associated H3K27me3 marks over the *Itch* promoter by ChIP assay. **(B)** Mouse peritoneal macrophages were infected with Mtb H37Rv for 24 h and assessed for the recruitment of PRMT5 and associated H4R3me2s marks over the *Itch* promoter by ChIP assay. **(C)** Mouse peritoneal macrophages were treated with PRMT5 inhibitor EPZ015666 for 1 h, followed by 24 h infection with Mtb H37Rv. Whole cell lysates were assessed for the expression of ITCH by immunoblotting. **(D)** Mouse peritoneal macrophages were transfected with NT or *Prmt5* siRNAs. Transfected cells were infected with Mtb H37Rv for 24 h and assessed for the expression of ITCH by immunoblotting. **(E)** Mouse peritoneal macrophages were infected with Mtb H37Rv for 24 h, whole cell lysates were immunoprecipitated with IgG control or PRMT5 antibodies and assessed for their interaction with YY1 by immunoblotting. **(F)** Mouse peritoneal macrophages were transfected with NT or *Yy1* siRNAs. Transfected cells were infected with Mtb H37Rv for 24 h and assessed for the recruitment of PRMT5 and associated H4R3me2s marks over the *Itch* promoter by ChIP assay. qRT-PCR data represents mean±S.E.M. and immunoblotting and ChIP data are representative of three independent experiments. NT, non-targeting; ChIP, chromatin immunoprecipitation. *p < 0.05; **, p < 0.01; *** p < 0.001; ****, p < 0.0001 (One way ANOVA; GraphPad Prism 6.0).

As the expression of ITCH was repressed upon mycobacterial infection, we sought to delineate the possible way in which YY1 could accomplish the same. Ample evidence has highlighted the association of YY1 with the Polycomb-group (PcG) proteins to bring about repression of target genes. In this context, upon binding to the DNA, YY1 could initiate PcG protein recruitment that results in concomitant histone deacetylation and methylation [32]. Thus, we surmised if YY1 could bring about repression of ITCH by recruiting members of the PcG proteins. A ChIP assay was performed to assess for the recruitment of the PcG group methyl transferase, EZH2 at the YY1 binding site on the promoter of *Itch*. It was observed that Mtb infection did not elicit an appreciable recruitment of EZH2 or its cognate methylation signature H3K27me3 at the YY1 binding sites on the promoter of *Itch* (Fig 4A). Based on this result, we construed that YY1 might interact with distinct epigenetic molecules other than EZH2 to bring about the repression of ITCH. Extensive review of literature revealed that PRMT1, an arginine methyl transferase could interact with YY1 to elicit the enhanced expression of genes [33]. It is important to note that PRMT1 effectuates asymmetric dimethylation in the H4R3 residue (H4R3me2a) to initiate the expression of genes. Interestingly, symmetric dimethylation at the same arginine residue of histone 4 (H4R3me2s) by PRMT5 brings about gene repression [34]. Thus, we conjectured if YY1 could interact with PRMT5 and catalyze the symmetric dimethylation (H4R3me2s) signature on the promoter region of *Itch* and arbitrate its repression. A ChIP assay confirmed the occupancy of PRMT5 and its cognate repressive methylation mark at the YY1-binding sites on the promoter of *Itch* (Fig 4B). We also observed that upon the perturbation of PRMT5 enzymatic activity with a specific inhibitor EPZ015666, Mtb-mediated diminished expression of ITCH was rescued in mouse peritoneal macrophages (Fig 4C). Besides, gene-specific knockdown of *Prmt5* in mouse peritoneal macrophages alleviated Mtb-mediated repression of ITCH during infection (Fig 4D). Further, immunoprecipitation analysis in murine macrophages revealed enhanced interaction between YY1 and PRMT5 upon mycobacterial infection (Fig 4E), implying the interaction between YY1 and PRMT5 on ITCH promoter. To conclusively implicate the significance of the association of YY1 and PRMT5 for the recruitment of the repressive methylation signature on the promoter of *Itch*, siRNA against *Yy1* was employed to selectively decrease the levels of *Yy1* in mouse peritoneal macrophages. Subsequently, it was observed that cells which were depleted of *Yy1* showed compromised recruitment of PRMT5 as well as the repressive methylation mark H4R3me2s on the *Itch* promoter (Fig 4F).

Together, these results suggest that Mtb infection mediates the repression of the concerned E3 ligase through the concerted action of YY1 and PRMT5.

### PRMT5 activity contributes to sustained lipid accumulation in Mtb-infected macrophages

**Fig 5:**
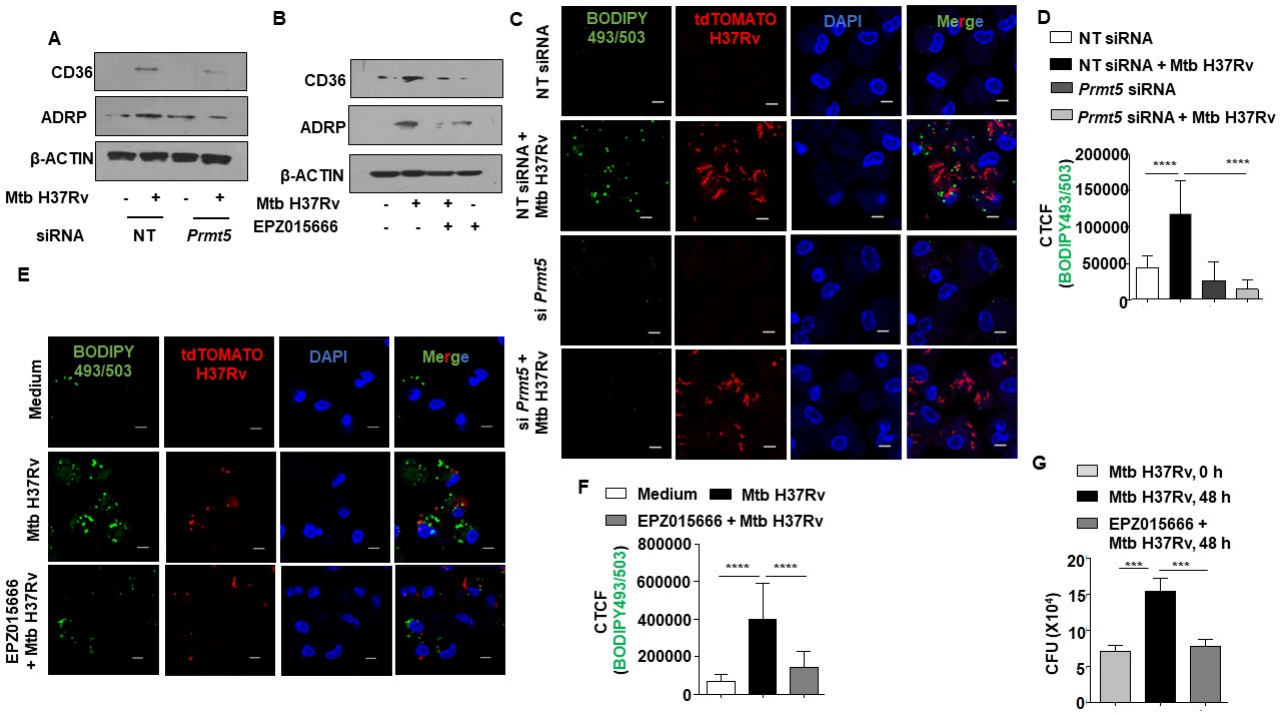
PRMT5 activity aids in the sustained accumulation of lipid droplets during Mtb infection. **(A)** Mouse peritoneal macrophages were transfected with NT or *Prmt5* siRNAs. Transfected cells were infected with Mtb H37Rv for 24 h and assessed for the expression of ADRP and CD36 by immunoblotting. **(B)** Mouse peritoneal macrophages were treated with PRMT5 inhibitor EPZ015666 for 1 h, followed by 24 h infection with Mtb H37Rv. Whole cell lysates were assessed for the expression of ADRP and CD36 by immunoblotting. **(C, D)** Mouse peritoneal macrophages were transfected with NT or *Prmt5* siRNAs. Transfected cells were infected with tdTomato Mtb H37Rv for 48 h and analyzed for lipid accumulation (BODIPY493/503) by confocal microscopy; **(C)** representative image and **(D)** respective quantification. **(E, F)** Mouse peritoneal macrophages were treated with PRMT5 inhibitor EPZ015666 for 1 h, followed by 48 h infection with tdTomato Mtb H37Rv and analyzed for lipid accumulation (BODIPY493/503) by confocal microscopy; **(E)** representative image and **(F)** respective quantification. **(G)** Mouse peritoneal macrophages were infected with Mtb H37Rv for 4 h. Extracellular bacteria were removed, and the infected cells were cultured in the presence or absence of PRMT5 inhibitor EPZ015666 for 48 h. Cells were lysed and plated on 7H11 to enumerate intracellular Mtb H37Rv burden. All immunoblotting and immunofluorescence data are representative of three independent experiments. NT, non-targeting; CTCF, corrected total cell fluorescence; CFU, colony forming units. *** p < 0.001; ****, p < 0.0001 (One way ANOVA; GraphPad Prism 6.0).

With the premise that YY1 and PRMT5 orchestrate the Mtb-mediated repression of ITCH, we assessed the role of PRMT5 in FM formation. We found that loss of PRMT5 restricted the Mtb-induced expression of ADRP and CD36, and consequently lipid accumulation (Fig 5A, 5C and 5D). In line, inhibition of PRMT5 enzymatic activity compromised the levels of CD36 and ADRP and the resultant FMs, thereby validating the essential role of PRMT5 in lipid accrual during mycobacterial infection (Fig 5B, 5E and 5F). Since accumulated lipids provide a favorable niche to the internalized mycobacteria [35], we evaluated mycobacterial survival in PRMT5 inhibitor-treated mouse peritoneal macrophages. Perturbation of PRMT5 enzyme activity in infected macrophages revealed a significant reduction in mycobacterial burden (Fig 5G), thereby indicating a compelling role of the PRMT5 inhibitor, EPZ015666, in reducing Mtb survival. Similarly, we observe compromised mycobacterial survival in cells knocked down for *Prmt5* or *Yy1* gene (S4C Fig). Notably, inhibition of PRMT5 did not affect bacterial uptake into host cells (S4A and S4B Fig). Further, it was our interest to investigate if deregulation in lipid levels contributed to the observed differences in mycobacterial survival upon NOTCH pathway or PRMT5 enzyme perturbation. In this context, oleic acid supplementation to mouse peritoneal macrophages resulted in enhanced mycobacterial survival in cells with compromised NOTCH pathway activation or PRMT5 enzymatic activity (S6 Fig), thereby substantiating our inferences on the specific role of PRMT5-mediated sustenance of lipids in mycobacterial survival within macrophages.

### Perturbation of PRMT5 activity aids in enhanced mycobacterial killing and resolution of granuloma-like lesions during infection

**Fig 6:**
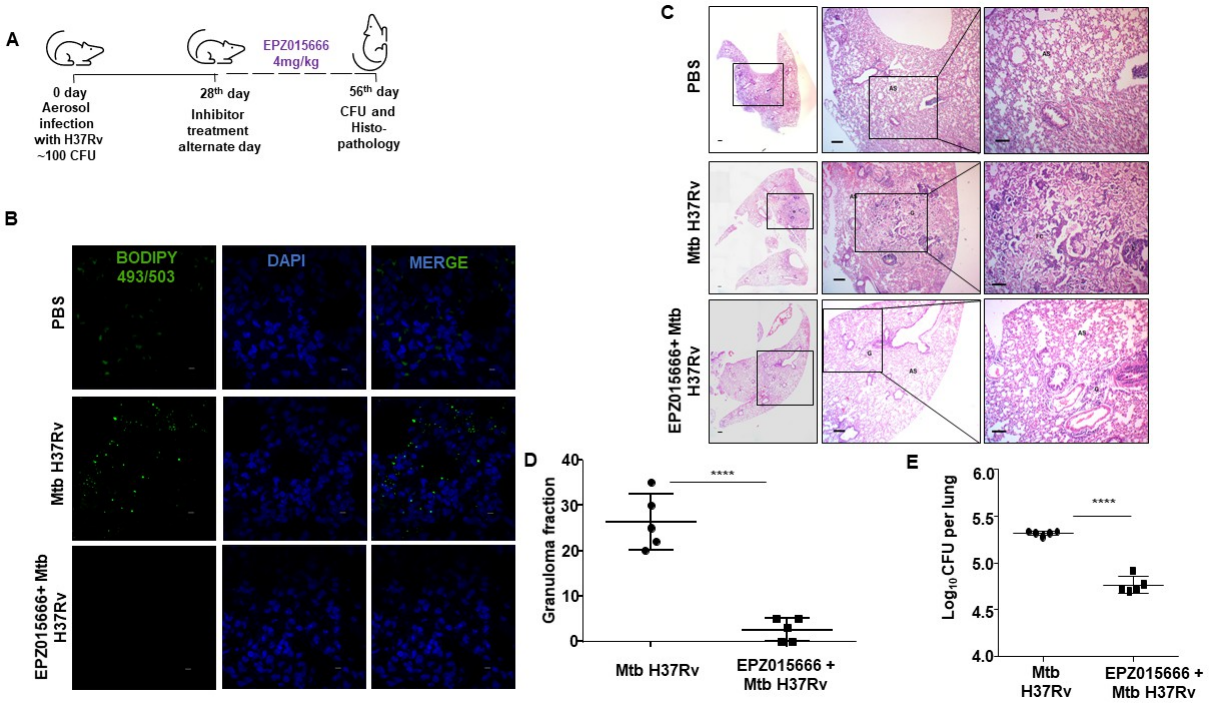
Inhibition of PRMT5 alleviates TB pathology. **(A)** Schematic representing the therapeutic regimen followed in a mouse model of Mtb infection. **(B-E)** BALB/c mice were infected with Mtb H37Rv and treated with PRMT5 inhibitor EPZ015666 (4mg/kg) as indicated in (A). (Number of mice per group = 5) **(B)** Lung cryosections were assessed for lipid accumulation by confocal microscopy (BODIPY493/503) (number of mice from which lung sections were assessed = 3 per group). **(C, D)** TB pathology (granulomatous lesions) in the lungs was analyzed by H and E staining; **(C)** representative images and **(D)** respective quantification (number of mice per group = 5). **(E)** Mycobacterial burden in the lungs of infected and PRMT5 inhibitor treated mice was enumerated by plating lung homogenates on 7H11 plates (number of mice per group = 5). Specific regions of the H and E-stained sections were zoomed by the pathologist for evaluation of granuloma fraction. Accordingly, the portions that have been zoomed are demarcated in the images; Magnification, 4X (left panel); 40X (middle panel); 100X (right panel); Scale bar for H and E images, 200 μm. Scale bar of immunofluorescence, 5μm. G, granulomatous lesion; AS, alveolar space, FC, foam cell; CFU, colony forming units. ****, p < 0.0001 (Student’s t-test; GraphPad Prism 9.0).

Having established the role of YY1-PRMT5 axis in mediating ITCH repression and consequently FM formation during Mtb infection, we employed an *in vivo* mouse model of TB (Fig 6A) to understand the effect of perturbing the signaling axis on mycobacterial burden and pulmonary pathology. Utilizing the specific pharmacological inhibitor, EPZ015666, we found that perturbation of PRMT5 enzyme activity compromised FM generation in the lungs of infected mice (Fig 6B). Further, the formation of hallmark TB granuloma-like lesions within the lungs of treated mice was significantly reduced as indicated by the H and E-stained lung sections and assessment of granuloma-like fraction within the infected lungs (Fig 6C and 6D). Corroborating our histological findings, we observed an appreciable decrease in mycobacterial burden in the lungs of Mtb-infected mice treated with PRMT5 inhibitor.

**Fig 7:**
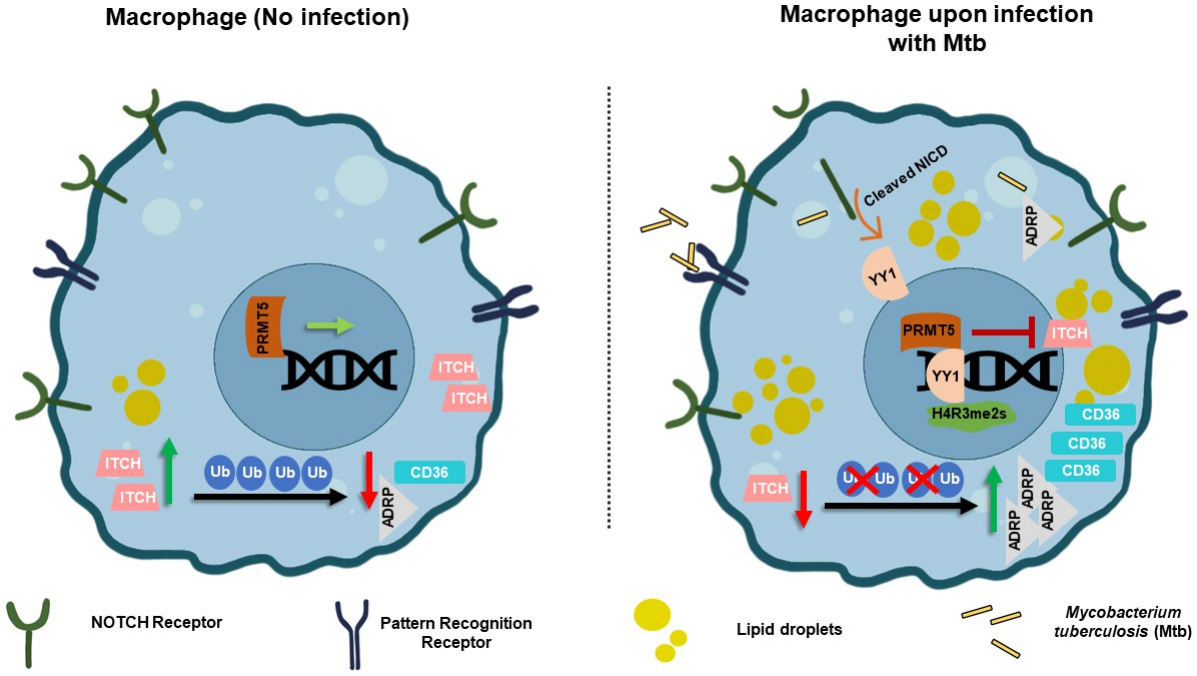
Model: Mycobacteria utilizes YY1-PRMT5 axis to repress ITCH and facilitate lipid accumulation.

Thus, we first report the YY1-PRMT5-dependent repression of the E3 ligase, ITCH, during Mtb infection. The reduction in the expression of ITCH contributed to enhanced lipid levels in infected cells, thereby aiding mycobacterial survival, both *in vitro* and *in vivo*. With these lines of evidence, we highlight the crucial contribution of NOTCH-YY1-PRMT5 axis in the pathogenesis of TB disease (Fig 7).

## Discussion

The enormous success of Mtb in continuing to affect human lives stems from its ability to persist within the body of the infected individual and attain adequate resistance against definitive drugs. FM formation has been attributed as a major development that aids pathogen survival within the hypoxic environment of the host cell. Mtb-induced FMs have been shown to harbor viable mycobacteria wherein it slowly acquires a dormant phenotype [36, 37]. Furthermore, FMs displayed enhanced protection from cell death [38] whilst also demonstrating a change to a more favorable inflammatory milieu for mycobacterial survival [4, 38]. Since several proteins have been implicated in Mtb-driven FM formation, it becomes pertinent to explore the regulatory mechanisms that contribute to the expression and stability of these proteins within the cells.

Ubiquitination is implicated in diverse cellular processes including protein degradation by the proteasome, cell cycle progression, transcriptional regulation, DNA repair and signal transduction [39]. Pathogenic species’ such as *Salmonella*, *Shigella* and *Legionella* secrete ubiquitin ligase-like effectors which are involved in the modulation of critical events of the host that subsequently aid in pathogen survival [40, 41]. Besides, mice deficient in the host E3 ligase gene, *Hectd3* could enhance host immune responses against bacteria such as *Francisella novicida*, *Mycobacterium bovis* (BCG), and *Listeria* and limit their dissemination [42]. In the current study, analysis of the expression of the NEDD4-family of E3 ligases revealed an appreciable decline in the expression of ITCH. While we established the role of ITCH repression in contributing to pro-mycobacterial FM formation, its role in modulating other cellular processes require further investigation. As mentioned earlier, the role of EGFR pathway in augmenting Mtb survival has been underscored previously [6, 43]. Besides, a separate report indicated a direct effect of ITCH on the stability of EGF tyrosine kinase receptor [44]. Thus, repression of ITCH could be yet another mechanism that contributes towards EGFR signaling activation during Mtb infection and consequently pathogen survival. Also, ITCH has been shown to regulate the turnover of distinct proteins involved in coordinating T cell immunity [45]. Altogether, additional consequences of Mtb-mediated downregulation of ITCH at the systemic scale other than its role in FM generation, would be promising avenues for future research.

Congruent to earlier reports [28], we found the expression of YY1 to be elevated upon virulent mycobacterial infection. In parallel studies, targeted knockdown of YY1 was shown to decrease the expression of several key effectors of lipid metabolism [46]. Besides, YY1has been reported to promote lipid accumulation in zebrafish liver [47]. Furthermore, YY1 has been implicated in different infectious scenarios. Notably, YY1 could regulate IFN-1 production [48], contribute to viral gene expression [49] and aid the integration of viral DNA into the host chromosomes [50] during discrete viral infections. Thus, it was of our interest to evaluate the role of YY1 in regulating events during mycobacterial infection, where we found its specific relevance in the formation of FMs, among other yet unexplored immune functions. Besides, our study designated a role for the NOTCH signaling pathway in the regulation of YY1.Available literature indicated the association between NOTCH pathway and YY1 upregulation as CBF-1 independent NOTCH signaling could modulate the gene expression of YY1 target genes [51]. An ancillary support for the role of NOTCH pathway could be derived from two reports wherein the juxtacrine signaling pathway was instrumental in *M*. *bovis*-mediated SOCS3 upregulation in macrophages on one hand [52], while on the other, YY1 was important for the elevated expression of SOCS3 in neuroinflammation and neuropathic pain [53].

Regulated histone methylation has a diverse role to play during several viral and bacterial infections, including TB [4, 18, 54, 55]. Here, we uncover the role of PRMT5 in mediating lipid accumulation through its close association with the transcriptional regulator, YY1. Histone methyl transferases, including those belonging to the arginine methyl transferase family (PRMTs) have been shown to associate with YY1. Precisely, PRMT1 and PRMT7 interact with YY1 to regulate the expression of specific genes [33, 56]. Besides, YY1 could transcriptionally activate PRMT5 and aid in proliferation and invasion in laryngeal cancer cells [57]. With our observation on the close association between PRMT5 and YY1, it would be worthwhile in future to study the association of YY1 with other distinct methyl transferases during mycobacterial infection.

In this study, we show that controlling the turnover of proteins associated with FMs via proteasomal degradation is important to aggregate lipids and that the downregulation of the E3 ubiquitin ligase ITCH by YY1-PRMT5 axis plays a primary role in this process. Besides, the lipids can also undergo turnover by distinct regulated processes such as enzymatic degradation (by lipases) and lipid-specific autophagy. Uncovering the contribution of each of these mechanisms would be useful in designing the most effective combination of drug targets against Mtb infection. Additionally, the effect of the use of the PRMT5 inhibitor in reducing Mtb burden requires further investigation. PRMT5 has been reported to negatively regulate cGAS-mediated antiviral responses [58]. It must be noted that available reports have alluded to the role of PRMT5 in regulating the alternative splicing of genes [59]. In this respect, Mtb infection has been shown to modulate alternate splicing events to program macrophage responses [60, 61], The reduced mycobacterial burden and granuloma-like lesions within the lungs of infected mice upon PRMT5 inhibitor administration is indicative of the beneficial effect of PRMT5 inhibition. This might be a result from a combination of LD reduction and the yet-unexplored modulation of alternative splicing and immune mechanisms. Thus, it would be imperative to evaluate the role of PRMT5 inhibitor in deregulating alternative splicing events and other discrete immune mechanisms during mycobacterial infection in future. Taken together, the current study unravels the implication of the interplay of specific signaling components and host epigenetic machinery in governing the pathogenesis of Mtb. Future studies involving gene-specific knockout animals would further add on to our knowledge of TB pathogenesis.

## Materials and methods

### Ethics statement

Experiments involving mice were carried out after the approval from Institutional Ethics Committee for animal experimentation, Indian Institute of Science (IISc), Bangalore, India (Approval Number: CAF/ETHICS/694/2019). The animal care and use protocol adhered were approved by national guidelines of the Committee for the Purpose of Control and Supervision of Experiments on Animals (CPCSEA), Government of India.

### Cells and mice

Four to six weeks old, male, and female BALB/c mice were utilized for all experiments. Mice were procured from The Jacksons Laboratory and maintained at the Central Animal Facility (CAF) in Indian Institute of Science (IISc) under 12-hour light and dark cycle. For *in vitro* experiments, mouse peritoneal macrophages were utilized. Briefly, mice were injected intraperitoneally with (4–8%) Brewer’s thioglycollate and peritoneal exudates were harvested in ice cold PBS after four days, seeded in tissue culture dishes. Adherent cells were utilized as peritoneal macrophages. RAW 264.7 mouse monocyte-like cell line was obtained from ATCC and the National Centre for Cell Sciences (NCCS), India. All cells were cultured in Dulbecco’s Modified Eagle Medium (DMEM, Gibco, Thermo Fisher Scientific) supplemented with 10% heat inactivated Fetal Bovine Serum (FBS, Gibco, Thermo Fisher Scientific) and maintained at 37°C in 5% CO_2_ incubator.

### Bacteria

Virulent strain of Mtb (Mtb H37Rv) was a kind research gift from Prof. Amit Singh, Department of Microbiology and Cell Biology, and Centre for Infectious Disease Research, IISc. Mycobacteria were cultured in Middlebrook 7H9 medium (Difco, USA) supplemented with 10% OADC (oleic acid, albumin, dextrose, catalase). Single-cell suspensions of mycobacteria were obtained by passing mid log phase culture through 23-, 28- and 30-gauge needle 10 times each and used for infecting mouse peritoneal macrophages or RAW 264.7 cells at multiplicity of infection 10. The studies involving virulent mycobacterial strains were carried out at the biosafety level 3 (BSL-3) facility at Centre for Infectious Disease Research (CIDR), IISc.

### Reagents and antibodies

All general chemicals and reagents were procured from Sigma-Aldrich/ Merck Millipore, HiMedia and Promega. Tissue culture plastic ware was purchased from Jet Biofil or Tarsons India Pvt. Ltd. and Corning Inc. siRNAs were obtained from Dharmacon as siGENOME SMART-pool reagents against *Yy1*, *Prmt5*, *Notch1*, *Itch*. Oleic acid, HRP-tagged anti-β-ACTIN (A3854), 4′,6-Diamidino-2-phenylindole dihydrochloride (DAPI) were procured from Sigma-Aldrich. Anti-cleaved NOTCH1, anti-ITCH, anti-YY1, anti- α-TUBULIN, anti-PRMT5, anti-EZH2, anti-H3K27me3 antibodies were procured from Cell Signaling Technology (USA). Anti-H4R3me2s antibody was sourced from Abcam. Anti-ADRP and anti-CD36 antibodies were procured from Santa Cruz Biotechnology (USA). Anti-LAMINB1 antibody was purchased from IMGENEX. HRP conjugated anti-rabbit IgG/anti-mouse IgG was obtained from Jackson ImmunoResearch (USA). Lipofectamine 3000 was purchased from Thermo Fisher Scientific. BODIPY 493/503 (4,4-Difluoro-1,3,5,7,8-Pentamethyl-4-Bora-3a,4a-Diaza-s-Indacene lipid stain was from Molecular Probes (Invitrogen/Thermo Fisher Scientific).

### Treatment with pharmacological reagents

Mouse peritoneal macrophages were pre-treated with the following reagents one hour prior to infection with Mtb H37Rv: GSI (Calbiochem, 10 μM); EPZ015666 (Tocris, 20 μM); Gefitinib (Cayman Chemicals, 20 μM); LY294002 (Calbiochem, 50 μM); IWP-2 (Calbiochem, 20 μM). MG132 (Sigma-Aldrich, 10 μM) was added 4 h. prior to harvest.

### Plasmids and constructs

NICD overexpression (OE) plasmid and β-galactosidase plasmids were received as kind gifts from Prof. Kumaralvel Somasundaram, (IISc, Bangalore). ITCH OE plasmid was procured from Addgene. HES1-luciferase plasmid was a kind gift from Prof. Ryoichiro Kageyama, Institute for Virus Research, Kyoto University.

### Transient transfection studies

RAW 264.7 macrophages were transfected with the indicated constructs (NICD OE, ITCH OE, HES1-luc.); or mouse peritoneal macrophages were transfected with 100 nM each of siGLO Lamin A/C, non-targeting siRNA or specific siRNAs with the help of Lipofectamine 3000 for 6 h; followed by 24 h recovery. 70–80% transfection efficiency was observed by counting the number of siGLO Lamin A/C positive cells in a microscopic field using fluorescence microscopy. Transfected cells were subjected to the required infections/ treatments for the indicated time points and processed for analyses.

### Luciferase assay

RAW 264.7 cells were transfected with HES1-luciferase and β-galactosidase plasmids using Lipofectamine 3000 for 6 h, followed by 24 h of recovery. subjected to the required infections/ treatments for the indicated time points and processed for analyses. Briefly, cells were harvested and lysed in reporter lysis buffer (Promega) and luciferase activity was assayed using luciferase assay reagent (Promega). The results were normalized for transfection efficiencies by assay of β-galactosidase activity.

### RNA isolation and quantitative real time PCR (qRT-PCR)

Treated samples were harvested in TRIzol (Sigma-Aldrich) and incubated with chloroform for phase separation. Total RNA was precipitated from the aqueous layer. Equal amount of RNA was converted into cDNA using First Strand cDNA synthesis kit (Applied Biological Materials Inc.). The cDNA thus obtained was used for SYBR Green (Thermo Fisher Scientific) based quantitative real time PCR analysis for the concerned genes. *Gapdh* was used as internal control gene. Primer pairs used for expression analyses are provided below (Table 1): Primers were synthesized and obtained from Eurofins Genomics Pvt. Ltd. (India).

**Table 1:** Primer sets used for gene expression analysis.

Genes	Forward primer (5’-3’)	Reverse primer (5’-3’)
*Gapdh*	GAGCCAAACGGGTCATCATCT	GAGGGGCCATCCACAGTCTT
*Nedd4*	GTGCCGATGAATGGGTTTGCTG	TCAACGCCATCAAAGCCCTGTG
*Nedd4-2*	TTGTCCTCTGACCCAATAGA	CAACTCTCCGATCTCCAATAAC
*Smurf1*	ACATCGTCAGGTGGTTCT	GGGCAGGTTGTCTGTATTG
*Smurf2*	TCAGTCCAGAGACCGAATAG	TCAGTCCAGAGACCGAATAG
*Wwp1*	CTTGCTCACTTCCGTTACTT	GCAAGGCCACCATAATC
*Wwp2*	CTCACCTACTTCCGCTTTATC	CACTCTTCCAGGTTGTTCTC
*Nedl1*	AACATCAAGTGTGCCCTATG	GGTGCTGGTTTCTTCTACTG
*Itch*	TGGGTAGTCTGACCATGAAATCT	GGGGTAACAATAACTGTGAGG
*Yy1*	CATCTGCACACCCACGGTCCCAGAGTCC	GCACACATAGGGCCTGTCTCCGGTATGG
*Hes1*	GAGAGGCTGCCAAGGTTTTTG	CACTGGAAGGTGACACTGCG
*Ifn a*	CTGAAGGACAGGAAGGACTTTG	AGAATGAGTCTAGGAGGGTTGT
*Ifnb*	ACTGGGTGGAATGAGACTATTG	CAGGCGTAGCTGTTGTACTT
*Socs3*	GCGAGAAGATTCCGCTGGTA	CCGTTGACAGTCTTCCGACAA

### Nuclear-cytoplasmic extraction

Cells were treated as indicated, harvested by centrifugation, and gently resuspended in ice-cold Buffer A (10 mM HEPES pH 7.9, 10 mM KCl, 0.1 mM EDTA, 0.1 mM EGTA, 1 mM DTT, and 0.5 mM PMSF). After incubation on ice for 15 min, cell membranes were disrupted with 10% NP-40 and the nuclear pellets were recovered by centrifugation at 13,226 × g for 15 min at 4°C. The supernatants from this step were used as cytosolic extracts. Nuclear pellets were lysed with ice-cold Buffer C (20 mM HEPES pH7.9, 0.4 M NaCl, 1 mM EDTA, 1 mM EGTA, 1 mM DTT, and 1 mM PMSF) and nuclear extracts were collected after centrifugation at 13,226 × g for 20 min at 4°C.

### Immunoblotting

Cells post treatment and/or infection were washed with 1X PBS. Whole cell lysate was prepared by lysing in RIPA buffer [50 mM Tris-HCl (pH 7.4), 1% NP-40, 0.25% sodium deoxycholate, 150 mM NaCl, 1 mM EDTA, 1 mM PMSF, 1 μg/mL each of aprotinin, leupeptin, pepstatin, 1 mM Na_3_VO_4_, 1 mM NaF] on ice for 30min. Total protein from whole cell lysates was estimated by Bradford reagent. Equal amount of protein was resolved on 12% SDS-PAGE and transferred onto PVDF membranes (Millipore) by semi-dry immunoblotting method (Bio-Rad). 5% non-fat dry milk powder in TBST [20 mM Tris-HCl (pH 7.4), 137 mM NaCl, and 0.1% Tween 20] was used for blocking nonspecific binding for 60 min. After washing with TBST, the blots were incubated overnight at 4°C with primary antibody diluted in TBST with 5% BSA. After washing with TBST, blots were incubated with anti-rabbit IgG secondary antibody conjugated to HRP antibody (111-035-045, Jackson ImmunoResearch) for 4h at 4°C. The immunoblots were developed with enhanced chemiluminescence detection system (Perkin Elmer) as per manufacturer’s instructions. For developing more than one protein at a particular molecular weight range, the blots were stripped off the first antibody at 60°C for 5 min using stripping buffer (62.5 mM Tris-HCl, with 2% SDS 100 mM 2-Mercaptoethanol), washed with 1X TBST, blocked; followed by probing with the subsequent antibody following the described procedure. β-ACTIN was used as loading control.

### Immunoprecipitation assay

Immunoprecipitation assays were carried out following a modified version of the protocol provided by Millipore, USA. Treated samples were washed in ice cold PBS and gently lysed in RIPA buffer. The cell lysates obtained were subjected to pre-clearing with BSA-blocked Protein A beads (Bangalore Genei, India) for 30 min at 4°C and slow rotation. The amount of protein in the supernatant was quantified and equal amount of protein was used for pull down from each treatment condition; using Protein A beads pre-conjugated with the antibody of interest or isotype control IgG antibody. After incubation of the whole cell lysates with the antibody-complexed beads for 4 h at 4°C on slow rotation, the pellet containing the bead-bound immune complexes were washed with RIPA buffer twice. The complexes were eluted by boiling the beads in Laemmli buffer for 10 min. The bead free samples were resolved by SDS-PAGE and the target interacting partners were identified by immunoblotting. Clean-Blot^™^ IP Detection Reagent (21230) was obtained from Thermo Scientific.

### Chromatin Immunoprecipitation (ChIP) assay

ChIP assays were carried out using a protocol provided by Upstate Biotechnology and Sigma-Aldrich with certain modifications. Briefly, treated samples were washed with ice cold 1X PBS and fixed with 3.6% formaldehyde for 15 min at room temperature followed by inactivation of formaldehyde with 125 mM glycine. Nuclei were lysed in 0.1% SDS lysis buffer [50 mM Tris-HCl (pH 8.0), 200 mM NaCl, 10 mM HEPES (pH 6.5), 0.1% SDS, 10 mM EDTA, 0.5 mM EGTA, 1 mM PMSF, 1 μg/ml of each aprotinin, leupeptin, pepstatin, 1 mM Na_3_VO_4_ and 1 mM NaF]. Chromatin was sheared using Bioruptor Plus (Diagenode, Belgium) at high power for 70 rounds of 30 sec pulse ON and 45 sec pulse OFF. Chromatin extracts containing DNA fragments with an average size of 500 bp were immunoprecipitated with the indicated antibodies or rabbit preimmune sera complexed with Protein A agarose beads (Bangalore Genei). Immunoprecipitated complexes were sequentially washed with Wash Buffer A, B and TE [Wash Buffer A: 50 mM Tris-HCl (pH 8.0), 500 mM NaCl, 1 mM EDTA, 1% Triton X-100, 0.1% Sodium deoxycholate, 0.1% SDS and protease/phosphatase inhibitors; Wash Buffer B: 50 mM Tris-HCl (pH 8.0), 1 mM EDTA, 250 mM LiCl, 0.5% NP-40, 0.5% Sodium deoxycholate and protease/phosphatase inhibitors; TE: 10 mM Tris-HCl (pH 8.0), 1 mM EDTA] and eluted in elution buffer [1% SDS, 0.1 M NaHCO_3_]. After treating the eluted samples with RNase A and Proteinase K, DNA was purified and precipitated using phenol-chloroform-ethanol method. Purified DNA was analyzed by quantitative real time RT-PCR. All values in the test samples were normalized to amplification of the specific gene in Input and IgG pull down and represented as fold change in modification or enrichment. All ChIP experiments were repeated at least three times. The list of primers is given below (Table 2): Primers were synthesized and obtained from Eurofins Genomics Pvt. Ltd. (India).

**Table 2:** Primer sets used for ChIP analysis.

S.No.	Gene	Forward primer (5’➔3’)	Reverse Primer (5’➔3’)
For YY1 binding			
1	*Itch* (Site1)	AACAGTGGTGGGCAGACATAGG	ATCCCAAAAACCCAAACCCA
2	*Itch* (Site2)	CCGAATTAATAGGCTGTGC	CCATCCTCCCAACTCAGAATC

### *In vitro* CFU analysis

Mouse peritoneal macrophages were infected with Mtb H37Rv at MOI 5 for 4 h. Post 4 h, the cells were thoroughly washed with PBS to remove any surface adhered bacteria and medium containing amikacin (0.2 mg/ml) was added for 2 h to deplete any extracellular mycobacteria. After amikacin treatment the cells thoroughly washed with PBS were taken for 0 h time point, and a duplicate set was maintained in antibiotic free medium for next 48 h along with respective inhibitors GSI and EPZ015666. Intracellular mycobacterial burden was enumerated by lysing macrophages with 0.06% SDS in 7H9 Middlebrook medium. Appropriate dilutions were plated on Middlebrook 7H11 agar plates supplemented with OADC (oleic acid, albumin, dextrose, catalase). Total colony forming units (CFUs) were counted after 21 days of plating.

### *In vivo* mouse model for TB and treatment with pharmacological inhibitor

BALB/c mice (n = 30) were infected with mid-log phase Mtb H37Rv, using a Madison chamber aerosol generation instrument calibrated to 100 CFU/animal. Aerosolized animals were maintained in securely commissioned BSL3 facility. Post 28 days of established infection, mice were administered eight intra-peritoneal doses (modified from [62]) of EPZ015666 (4mg/kg) every alternate day over 28 days. On 56^th^ day post inhibitor treatment, mice were sacrificed, the left lung lobe was homogenized in sterile PBS, serially diluted, and plated on 7H11 agar containing OADC to quantify CFU. Upper right lung lobes were fixed in formalin, and processed for hematoxylin and eosin staining, or immunofluorescence analyses. Also, specific lobes from the lungs of mice were homogenized for the extraction of RNA and protein.

### Hematoxylin and Eosin staining

Microtome sections (5 μm) were obtained from formalin-fixed, paraffin-embedded mouse lung tissue samples using Leica RM2245 microtome. Deparaffinized and rehydrated sections were subjected to Hematoxylin staining followed by Eosin staining as per manufacturer instructions. After dehydrating, sections were mounted using permount. Sections were kept for drying overnight and handed over to consultant pathologist for blinded analyses. The pathologists have performed the analysis based on the article by Palanisamy *et al*. Tuberculosis (Edinb). 2008 [63].

### Cryosection preparation

The excised lung tissue portions were fixed in 4% paraformaldehyde solution. Subsequently, the tissues were kept in 30% sucrose solution. The fixed lung pieces were placed in the optimal cutting temperature (OCT) media (Jung, Leica). Cryosections of 10 μm were prepared using Leica CM 1510 S or Leica CM 3050 S cryostat and then stored at -80°C.

### Immunofluorescence

Cells were fixed with 3.6% formaldehyde for 30 min. at room temperature. Fixed samples were blocked with 2% BSA in PBST (containing 0.02% saponin) for 1 h. After blocking, samples were stained with the indicated antibodies at 4°C overnight, followed by incubation with DyLight 488-, Alexa 549- or Alexa 643-conjugated secondary antibodies for 2 h and nuclei were stained with DAPI. The samples were mounted on glycerol. Confocal images were taken with Zeiss LSM 710 Meta confocal laser scanning microscope (Carl Zeiss AG, Germany) using a plan-Apochromat 63X/1.4 Oil DIC objective (Carl Zeiss AG, Germany) and images were analyzed using ZEN 2009 software.

### Lipid droplet staining

Lipid droplets were stained using neutral lipid dye BODIPY 493/503 (Invitrogen). The cells/cryosection tissues were fixed with 3.6% formaldehyde for 30 min. After 3 washes with PBS, the cells/cryosection tissues were stained with 10 μg/mL BODIPY 493/503 for 30 min in the dark. Subsequently, the cells were washed, and nuclei were stained with DAPI. After washing with PBS, samples were mounted on glycerol and visualized and analyzed by confocal microscopy. For quantitative estimation of the results, at least 200 cells from different fields were analyzed. For the Mean Fluorescence Intensity (MFI) analysis, ImageJ was utilized to calculate the maximum intensity projections of the Z-stacks. Using free hand selection tool, cells were selected to measure the area-integrated intensity and mean grey value. The area around the cells without fluorescence was used to calculate the background values. Corrected Total Cell Fluorescence (CTCF) was calculated using the following formula: CTCF = Integrated intensity–(area of selected cell X Mean florescence of background reading).

### MTT assay

Mouse peritoneal macrophages were transfected or treated with the desired siRNA or inhibitors and cultured for 48 h. Thereafter, 3-(4, 5-dimethylthiazol-2-yl)-2, 5-diphenyltetrazolium bromide (MTT; 5 mg/mL) was added to the medium. MTT is a tetrazolium salt that is converted by living cells into blue formazan crystals. After incubating for 3h, the medium is removed from the wells and 200 μL dimethyl sulfoxide was added to dissolve formazan crystals. Thereafter, the absorbance was measured at 570 nm in an enzyme-linked immunosorbent assay reader. For all cytotoxicity assays, the viability of control cells (treated with DMSO or transfected with non-targeting, NT siRNA) was made to 100% and relative viability of test conditions was calculated as a percentage.

### Statistical analysis

Levels of significance for comparison between samples were determined by the student’s t-test and one-way ANOVA followed by Tukey’s multiple-comparisons. The data in the graphs are expressed as the mean ± S.E. for the values from at least 3 or more independent experiments and P values < 0.05 were defined as significant. GraphPad Prism software (6.0, 9.0 versions, GraphPad Software, USA) was used for all the statistical analyses.

## Supporting information

S1 Fig ITCH is downregulated during mycobacterial infection. **(A)** Mouse peritoneal macrophages were infected with Mtb H37Rv for the indicated time points and assessed for the transcript levels of NEDD family E3 ubiquitin ligases. **(B)** BALB/c mice were aerosol-infected with Mtb H37Rv for 28 days. Transcript levels of NEDD family E3 ubiquitin ligases was analyzed in the lung homogenates of uninfected and infected mice by qRT-PCR, (number of mice per group = 4). qRT-PCR data represents mean±S.E.M. from three independent experiments. *, p < 0.05; **, p < 0.01; *** p < 0.001 (Student’s t-test; GraphPad Prism 6.0).
(TIF)

S2 Fig Validation of YY1 siRNA. Mouse peritoneal macrophages were transfected with NT or *Yy1* siRNA. Transfected cells were infected with Mtb H37Rv for 24 h and assessed for the expression of YY1 by immunoblotting. Immunoblotting data is representative of three independent experiments. NT, non-targeting. β-ACTIN was utilized as loading control.
(TIF)

S3 Fig NOTCH signaling is upregulated upon mycobacterial infection. **(A)** Mouse peritoneal macrophages were infected with Mtb H37Rv for 1 h. Whole cell lysates were assessed for NICD expression by immunoblotting. **(B)** Mouse peritoneal macrophages were infected with Mtb H37Rv for 24 h and assessed for the expression of NOTCH target gene *Hes1* by qRT-PCR. **(C)** RAW264.7 macrophages were transiently transfected with HES1-reporter luciferase and the transfected cells were infected with Mtb H37Rv for 24 h, followed by assessment of luciferase counts using luminometer. Immunoblotting data is representative of three independent experiments; qRT-PCR and luciferase data represents mean±S.E.M of three independent experiments. NICD, NOTCH intracellular domain; Med, Medium. β-ACTIN was utilized as loading control. *, p < 0.05; *** p < 0.001 (Student’s t-test; GraphPad Prism 6.0).
(TIF)

S4 Fig Perturbation of NOTCH, PRMT5 or YY1 does not affect mycobacterial uptake in macrophages. **(A)** Mouse peritoneal macrophages were pre-treated with GSI (NOTCH pathway inhibitor) or EPZ015666 (PRMT5 inhibitor) for 1 h and infected with Mtb H37Rv for 4 h. Extracellular bacteria were removed, and cells were lysed and plated on 7H11 agar to enumerate internalized mycobacteria. **(B)** Mouse peritoneal macrophages were transfected with NT, *Yy1* or *Prmt5* siRNAs. Transfected cells were infected with Mtb H37Rv for 4 h. Extracellular bacteria were removed, and cells were lysed and plated on 7H11 agar to enumerate internalized mycobacteria. **(C)** Mouse peritoneal macrophages were transfected with NT, *Yy1* or *Prmt5* siRNAs. Transfected cells were infected with Mtb H37Rv for 4 h. Extracellular bacteria were removed, and the infected cells were cultured for 48 h. Cells were lysed and plated on 7H11 agar to enumerate intracellular Mtb H37Rv burden. NT, non-targeting; CFU, colony forming units. ns, non-significant; ****, p < 0.0001 (Student’s t-test; GraphPad Prism 9.0).
(TIF)

S5 Fig Validation of PRMT5 siRNA. Mouse peritoneal macrophages were transfected with NT or *Prmt5* siRNA. Transfected cells were infected with Mtb H37Rv for 24 h and assessed for the expression of PRMT5 by immunoblotting. Immunoblotting data is representative of three independent experiments. NT, non-targeting. β-ACTIN was utilized as loading control.
(TIF)

S6 Fig Oleic acid supplementation enhances mycobacterial survival in cells wherein PRMT5 and NOTCH pathway activation is compromised. Mouse peritoneal macrophages were pre-treated with GSI (NOTCH pathway inhibitor) and EPZ015666 (PRMT5 inhibitor) for 1 h and then infected with Mtb H37Rv. Extracellular bacteria were removed and subsequently, a set of infected cells were supplemented with oleic acid. Post 48h of infection, cells were lysed and plated on 7H11 agar to assess mycobacterial burden. ***, p < 0.001; ****, p < 0.0001 (one-way ANOVA, GraphPad Prism9.0).
(TIF)

S7 Fig Repression of ITCH (*Itch*) does not regulate ADRP (*Plin2*) and CD36 (*Fat*) *at the transcript level*. ***(A****)* Mouse peritoneal macrophages were transfected with NT *or Itch* siRNAs. Transfected cells were assessed for the expression of *Plin2* and *Fat* by qRT-PCR. **(B)** Mouse peritoneal macrophages were transfected with NT or *Itch* siRNAs. Transfected cells were assessed for the expression of *Itch* by qRT-PCR to verify the knockdown of *Itch*. qRT-PCR data represents mean±S.E.M. from three independent experiments. ns, non-significant; ****, p < 0.0001 (Student’s t-test; GraphPad Prism 6.0).
(TIF)

S8 Fig Effect of *Yy1* knockdown on the expression of *Ifna*, *Ifnb* and *Socs3*. **(A, B, C) (A)** Mouse peritoneal macrophages were transfected with NT or *Yy1* siRNAs. Transfected cells were subsequently infected with Mtb H37Rv for 24h and assessed for the expression of *Ifna* and *Ifnb* **(A)**, *Socs3* **(B)** and *Yy1* **(C)** by qRT-PCR. qRT-PCR data represents mean±S.E.M. from three independent experiments. NT, non-targeting; ns, non-significant; ***, p < 0.001; ****, p < 0.0001 (Two-way ANOVA; GraphPad Prism 9.0).
(TIF)

S9 Fig Relative expression of ITCH in the lungs of mice infected with Mtb H37Rv. BALB/c mice were infected with Mtb H37Rv for 28 days. Lung cryosections were assessed for the expression of ITCH and macrophage marker (F4/80) by confocal microscopy (Lung cryosections from three mice for each group was analyzed). Scale bar for immunofluorescence, 5μm.
(TIF)

S10 Fig Relative expression of YY1 in the lungs of mice infected with Mtb H37Rv. BALB/c mice were infected with Mtb H37Rv for 28 days. Lung cryosections were assessed for the expression of YY1 and macrophage marker (F4/80) by confocal microscopy (Lung cryosections from three mice for each group was analyzed). Scale bar for immunofluorescence, 5μm.
(TIF)

S11 Fig Effect of the perturbation of NOTCH pathway, PRMT5 and YY1 depletion on macrophage cell viability. **(A)** Mouse peritoneal macrophages were treated with PRMT5 inhibitor- EPZ015666 (20 μM) and NOTCH pathway inhibitor- GSI (10μM) for 48 h and percent viability of the cells was determined by MTT assay. **(B)** Mouse peritoneal macrophages were transfected with NT, *Yy1*, or *Prmt5* siRNAs. Transfected cells were cultured in the medium for 48 h after recovery and subsequently assessed for cell viability by MTT assay. MTT, (3-(4,5-dimethylthiazol-2-yl)-2,5-diphenyltetrazolium bromide); NT, non-targeting; ns, non-significant (Student’s t-test; GraphPad Prism 9.0).
(TIF)
