# Supplementary figures and images for: PRMT5 epigenetically regulates the E3 ubiquitin ligase ITCH to influence lipid accumulation during mycobacterial infection
Source: PLoS Pathog. 2022 Jun 3;18(6):e1010095. doi: 10.1371/journal.ppat.1010095 (PMC9200362; doi:10.1371/journal.ppat.1010095)

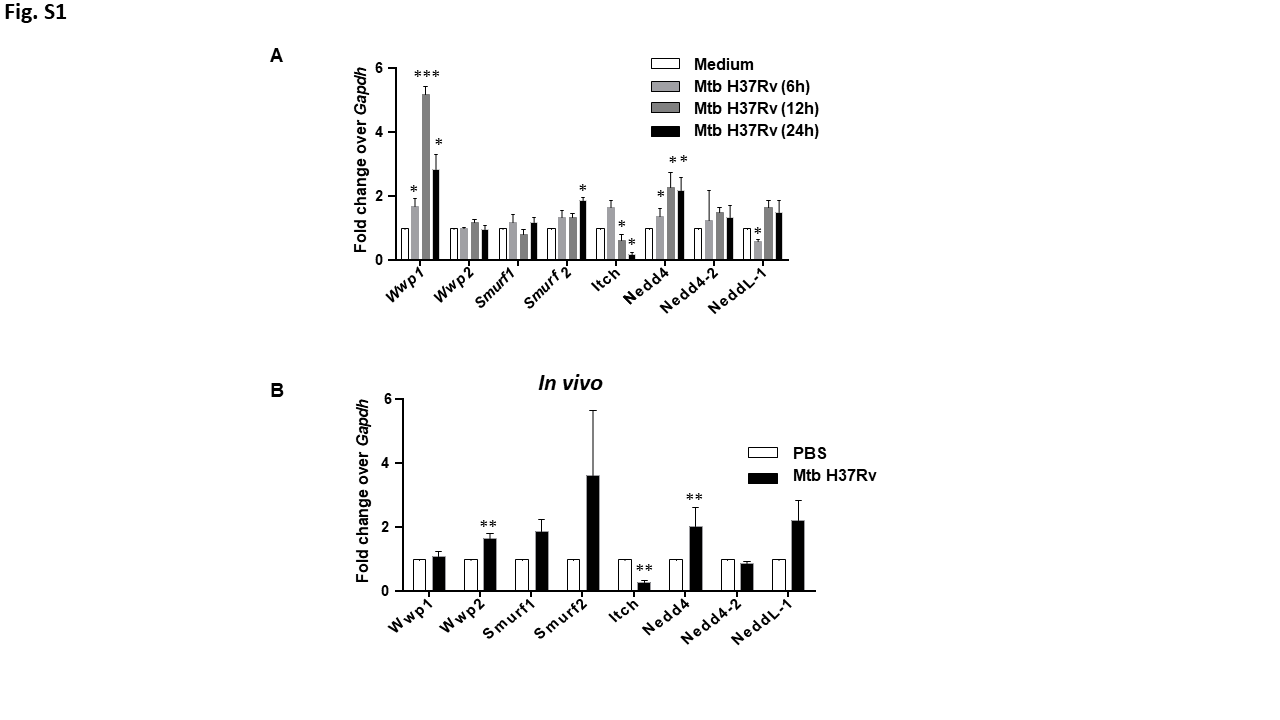

Supplement: S1 Fig — (A) Mouse peritoneal macrophages were infected with Mtb H37Rv for the indicated time points and assessed for the transcript levels of NEDD family E3 ubiquitin ligases. (B) BALB/c mice were aerosol-infected with Mtb H37Rv for 28 days. Transcript levels of NEDD family E3 ubiquitin ligases was analyzed in the lung homogenates of uninfected and infected mice by qRT-PCR, (number of mice per group = 4). qRT-PCR data represents mean±S.E.M. from three independent experiments. *, p < 0.05; **, p < 0.01; *** p < 0.001 (Student’s t-test; GraphPad Prism 6.0). (TIF) [file ppat.1010095.s001.TIF]

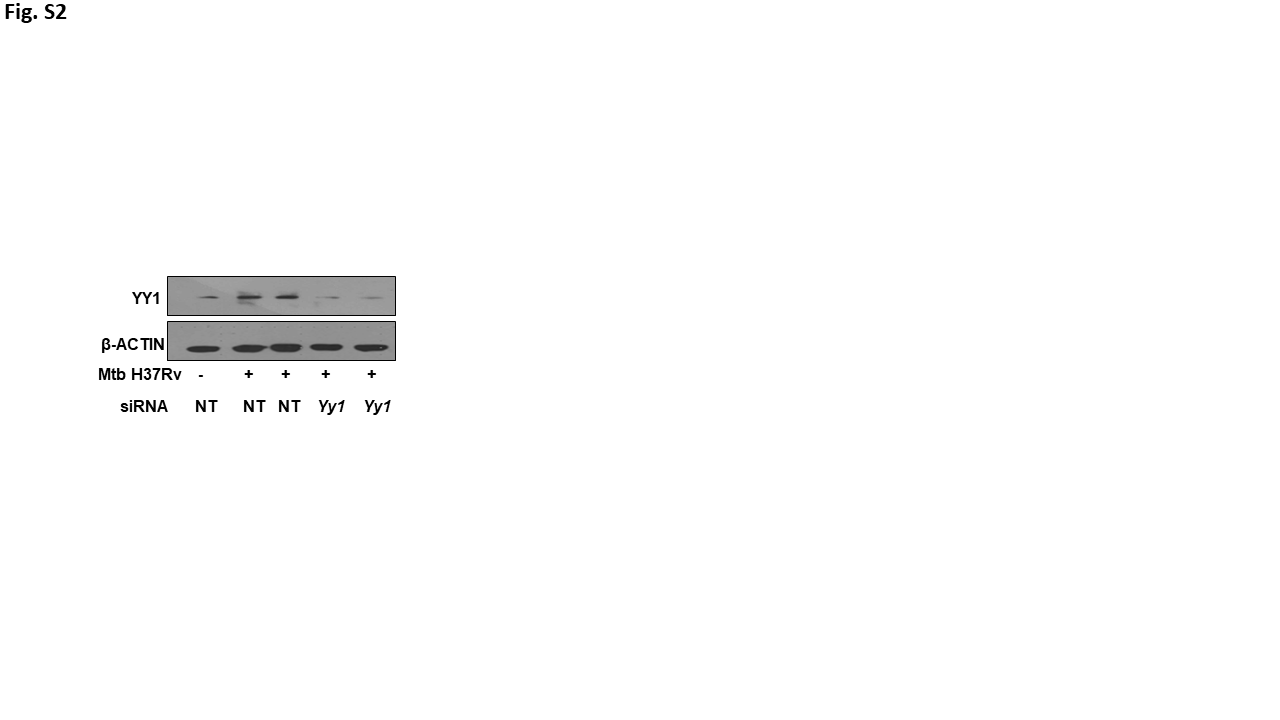

Supplement: S2 Fig — Mouse peritoneal macrophages were transfected with NT or Yy1 siRNA. Transfected cells were infected with Mtb H37Rv for 24 h and assessed for the expression of YY1 by immunoblotting. Immunoblotting data is representative of three independent experiments. NT, non-targeting. β-ACTIN was utilized as loading control. (TIF) [file ppat.1010095.s002.TIF]

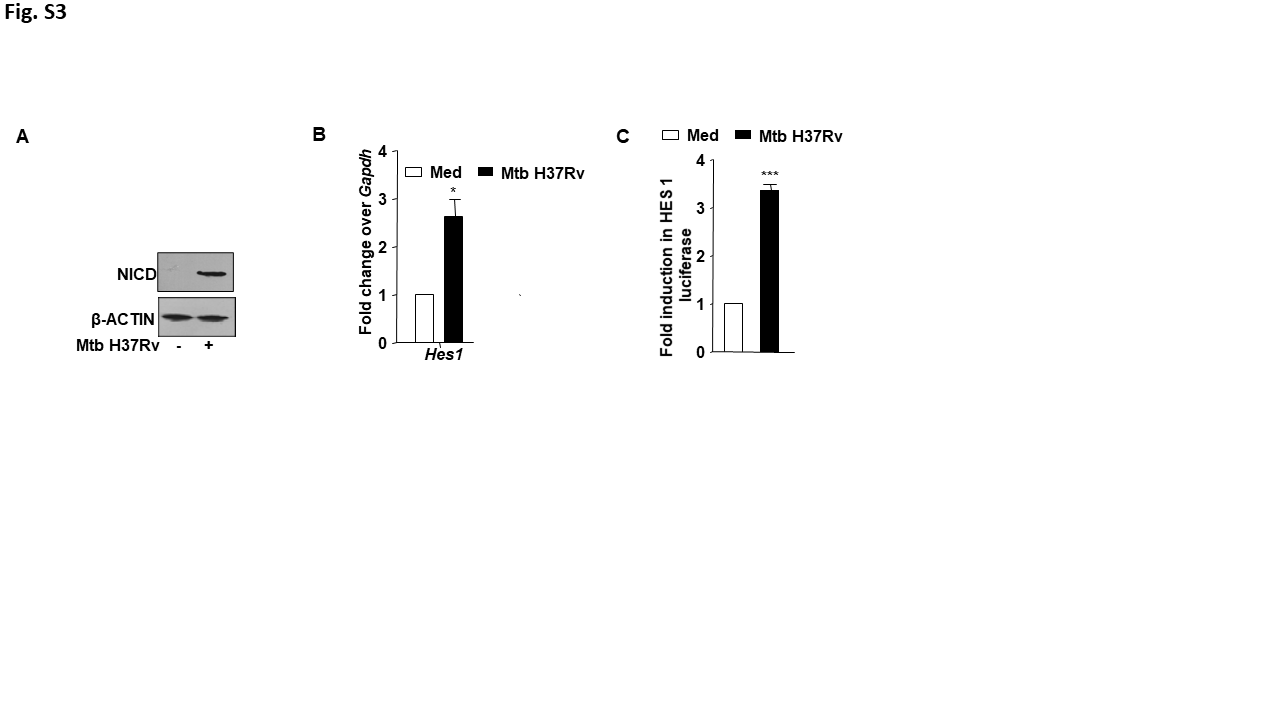

Supplement: S3 Fig — (A) Mouse peritoneal macrophages were infected with Mtb H37Rv for 1 h. Whole cell lysates were assessed for NICD expression by immunoblotting. (B) Mouse peritoneal macrophages were infected with Mtb H37Rv for 24 h and assessed for the expression of NOTCH target gene Hes1 by qRT-PCR. (C) RAW264.7 macrophages were transiently transfected with HES1-reporter luciferase and the transfected cells were infected with Mtb H37Rv for 24 h, followed by assessment of luciferase counts using luminometer. Immunoblotting data is representative of three independent experiments; qRT-PCR and luciferase data represents mean±S.E.M of three independent experiments. NICD, NOTCH intracellular domain; Med, Medium. β-ACTIN was utilized as loading control. *, p < 0.05; *** p < 0.001 (Student’s t-test; GraphPad Prism 6.0). (TIF) [file ppat.1010095.s003.TIF]

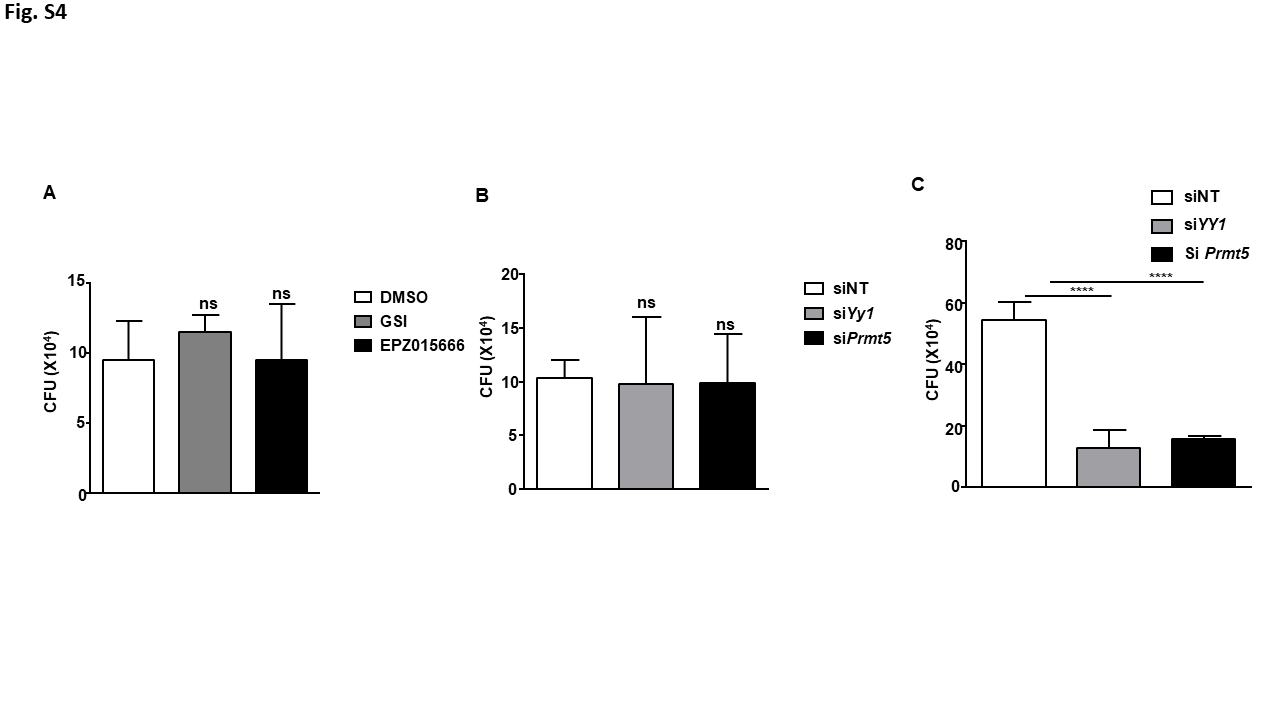

Supplement: S4 Fig — (A) Mouse peritoneal macrophages were pre-treated with GSI (NOTCH pathway inhibitor) or EPZ015666 (PRMT5 inhibitor) for 1 h and infected with Mtb H37Rv for 4 h. Extracellular bacteria were removed, and cells were lysed and plated on 7H11 agar to enumerate internalized mycobacteria. (B) Mouse peritoneal macrophages were transfected with NT, Yy1 or Prmt5 siRNAs. Transfected cells were infected with Mtb H37Rv for 4 h. Extracellular bacteria were removed, and cells were lysed and plated on 7H11 agar to enumerate internalized mycobacteria. (C) Mouse peritoneal macrophages were transfected with NT, Yy1 or Prmt5 siRNAs. Transfected cells were infected with Mtb H37Rv for 4 h. Extracellular bacteria were removed, and the infected cells were cultured for 48 h. Cells were lysed and plated on 7H11 agar to enumerate intracellular Mtb H37Rv burden. NT, non-targeting; CFU, colony forming units. ns, non-significant; ****, p < 0.0001 (Student’s t-test; GraphPad Prism 9.0). (TIF) [file ppat.1010095.s004.TIF]

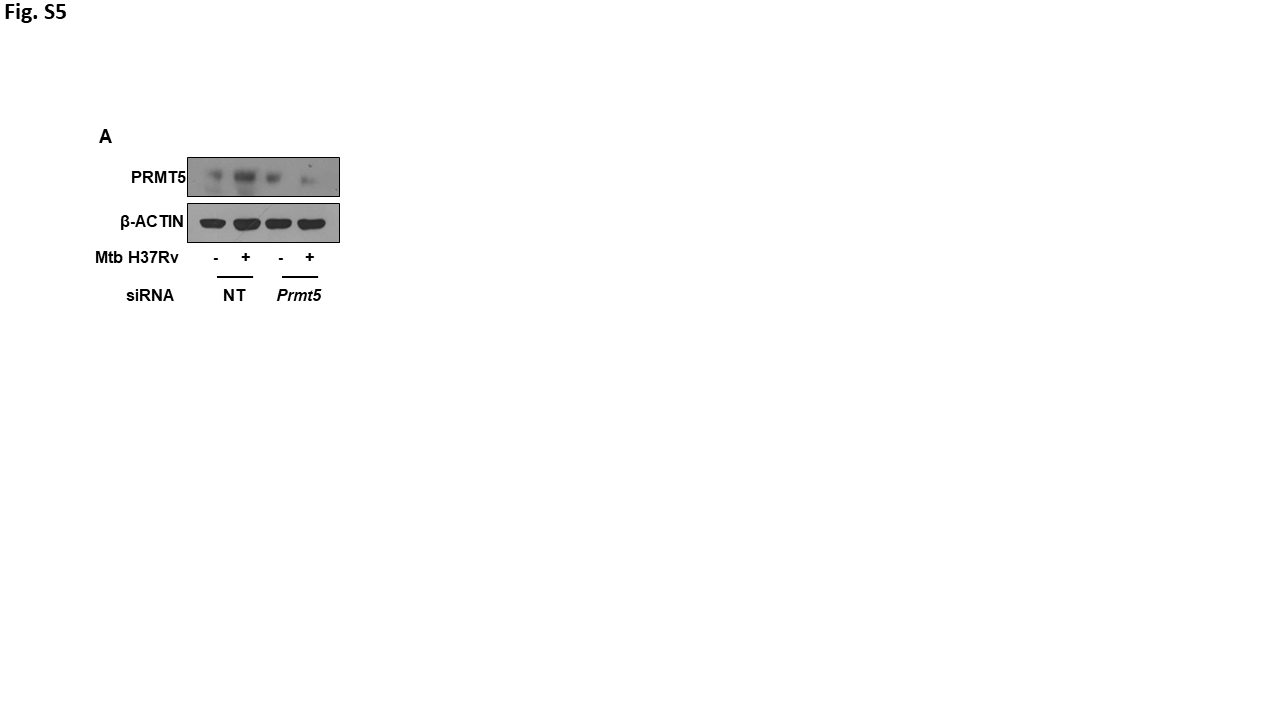

Supplement: S5 Fig — Mouse peritoneal macrophages were transfected with NT or Prmt5 siRNA. Transfected cells were infected with Mtb H37Rv for 24 h and assessed for the expression of PRMT5 by immunoblotting. Immunoblotting data is representative of three independent experiments. NT, non-targeting. β-ACTIN was utilized as loading control. (TIF) [file ppat.1010095.s005.TIF]

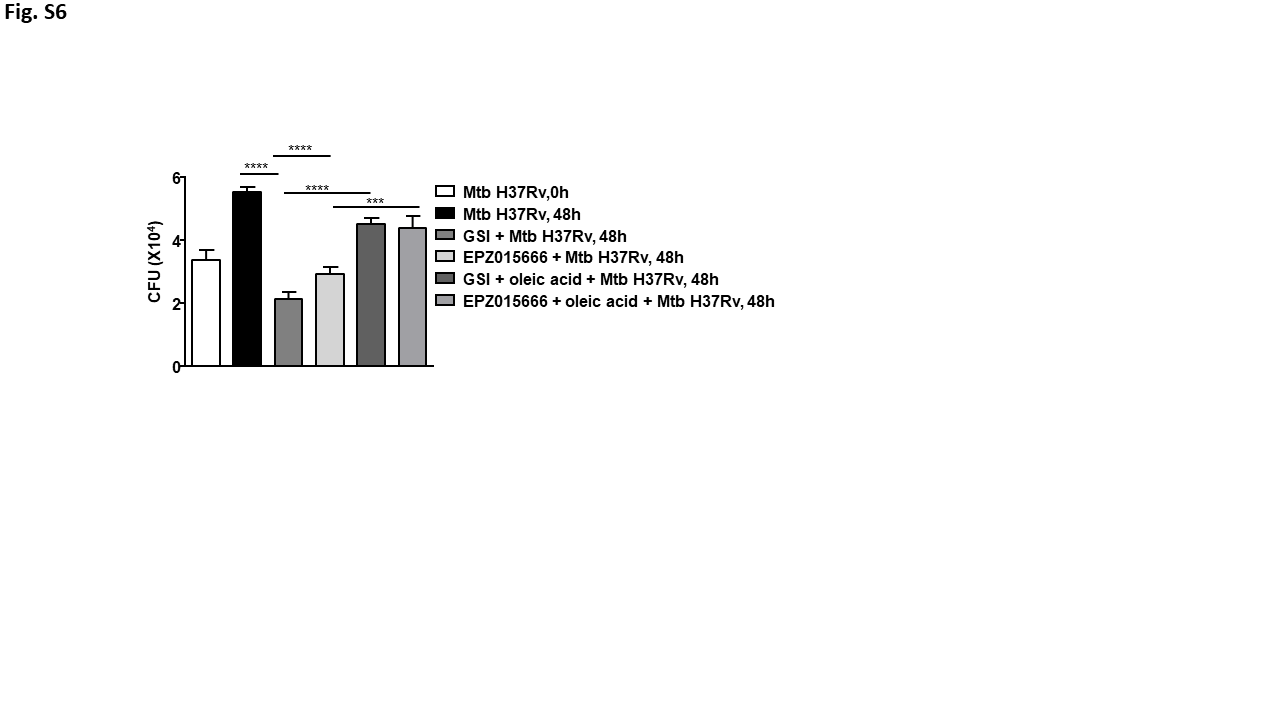

Supplement: S6 Fig — Mouse peritoneal macrophages were pre-treated with GSI (NOTCH pathway inhibitor) and EPZ015666 (PRMT5 inhibitor) for 1 h and then infected with Mtb H37Rv. Extracellular bacteria were removed and subsequently, a set of infected cells were supplemented with oleic acid. Post 48h of infection, cells were lysed and plated on 7H11 agar to assess mycobacterial burden. ***, p < 0.001; ****, p < 0.0001 (one-way ANOVA, GraphPad Prism9.0). (TIF) [file ppat.1010095.s006.TIF]

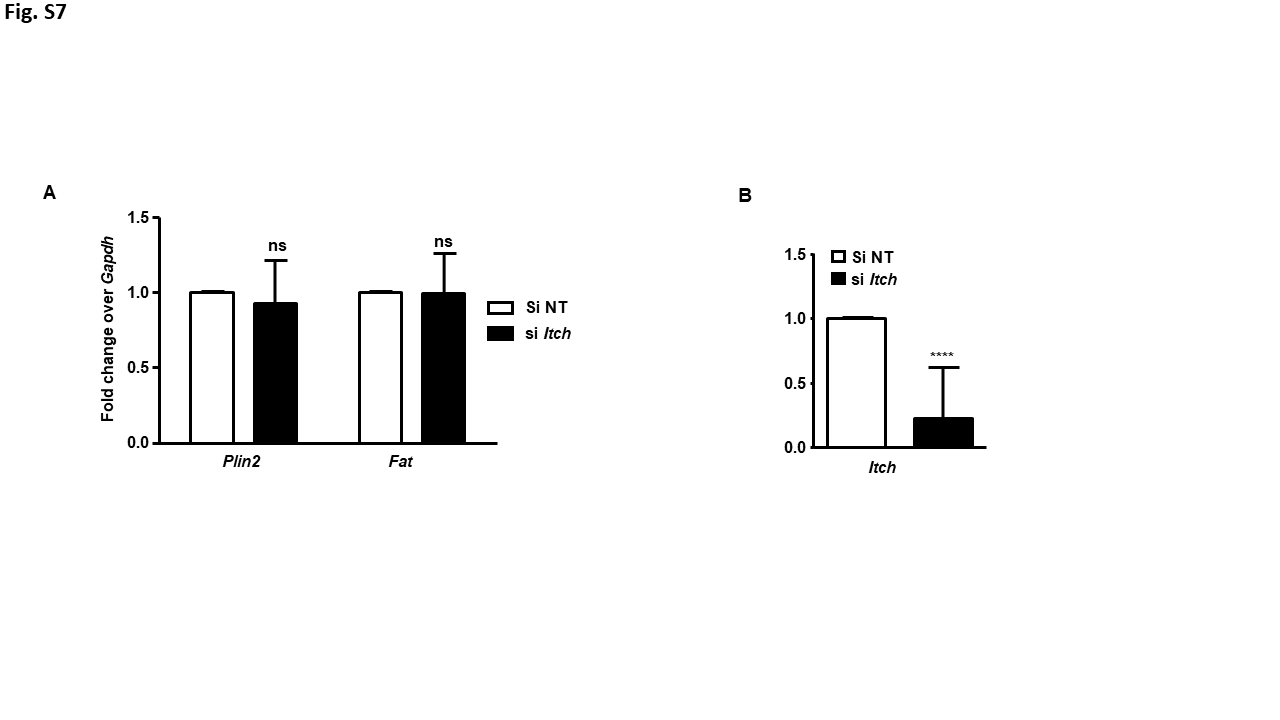

Supplement: S7 Fig — (A) Mouse peritoneal macrophages were transfected with NT or Itch siRNAs. Transfected cells were assessed for the expression of Plin2 and Fat by qRT-PCR. (B) Mouse peritoneal macrophages were transfected with NT or Itch siRNAs. Transfected cells were assessed for the expression of Itch by qRT-PCR to verify the knockdown of Itch. qRT-PCR data represents mean±S.E.M. from three independent experiments. ns, non-significant; ****, p < 0.0001 (Student’s t-test; GraphPad Prism 6.0). (TIF) [file ppat.1010095.s007.TIF]

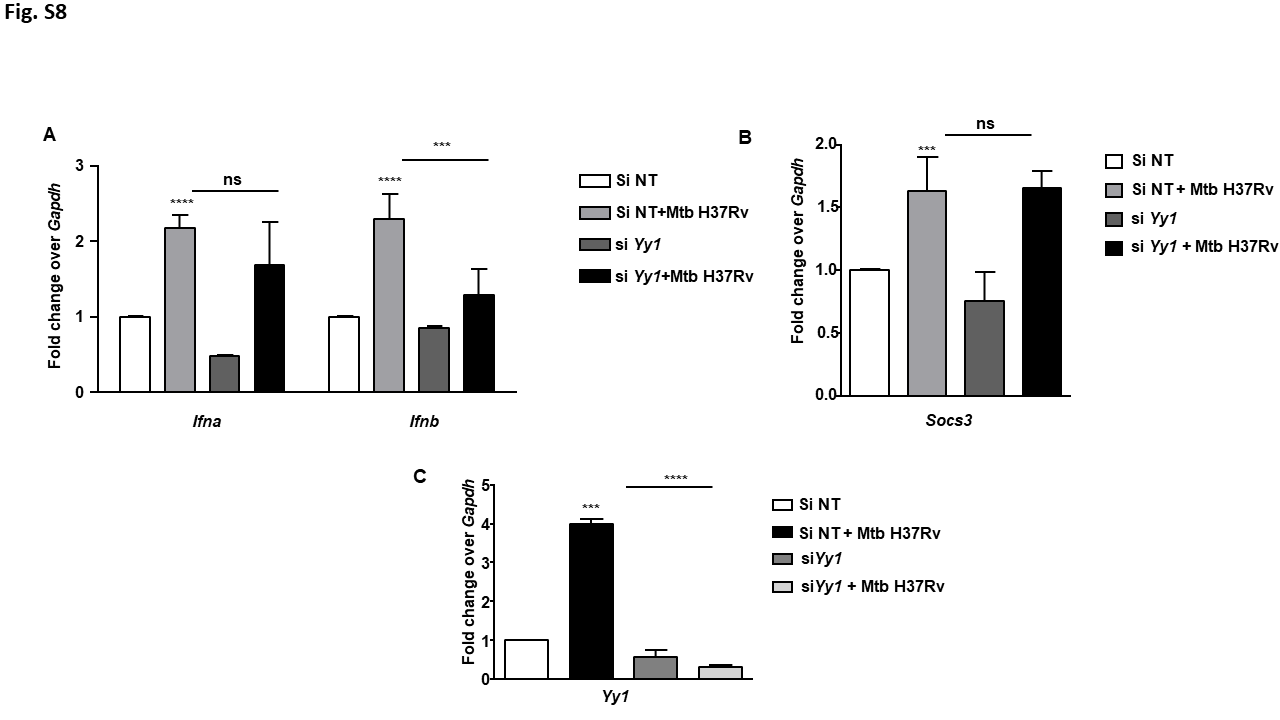

Supplement: S8 Fig — (A, B, C) (A) Mouse peritoneal macrophages were transfected with NT or Yy1 siRNAs. Transfected cells were subsequently infected with Mtb H37Rv for 24h and assessed for the expression of Ifna and Ifnb (A), Socs3 (B) and Yy1 (C) by qRT-PCR. qRT-PCR data represents mean±S.E.M. from three independent experiments. NT, non-targeting; ns, non-significant; ***, p < 0.001; ****, p < 0.0001 (Two-way ANOVA; GraphPad Prism 9.0). (TIF) [file ppat.1010095.s008.TIF]

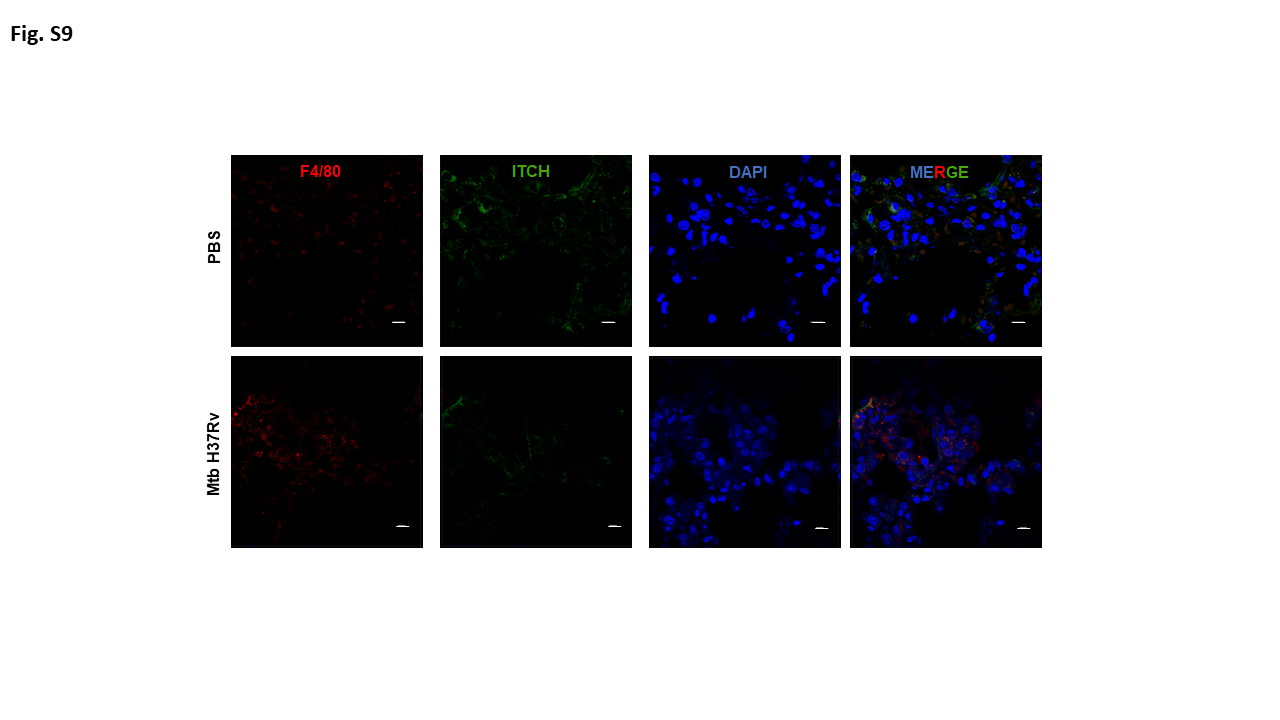

Supplement: S9 Fig — BALB/c mice were infected with Mtb H37Rv for 28 days. Lung cryosections were assessed for the expression of ITCH and macrophage marker (F4/80) by confocal microscopy (Lung cryosections from three mice for each group was analyzed). Scale bar for immunofluorescence, 5μm. (TIF) [file ppat.1010095.s009.TIF]

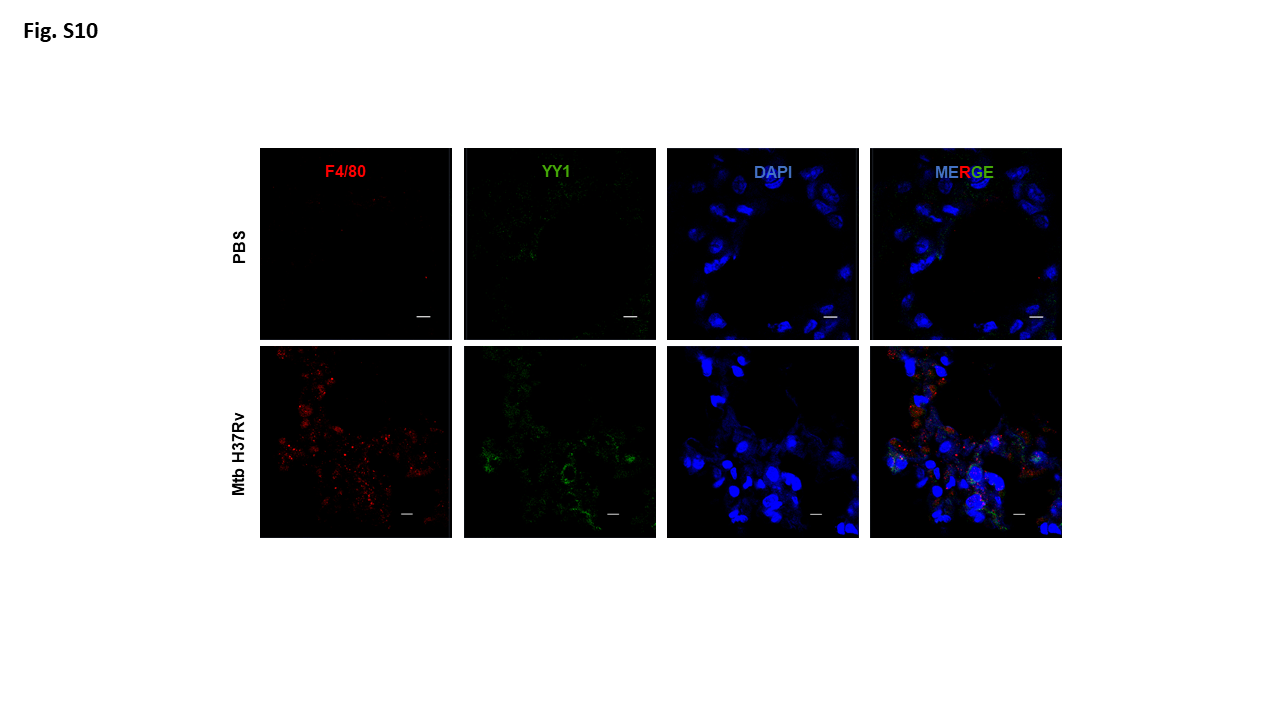

Supplement: S10 Fig — BALB/c mice were infected with Mtb H37Rv for 28 days. Lung cryosections were assessed for the expression of YY1 and macrophage marker (F4/80) by confocal microscopy (Lung cryosections from three mice for each group was analyzed). Scale bar for immunofluorescence, 5μm. (TIF) [file ppat.1010095.s010.TIF]

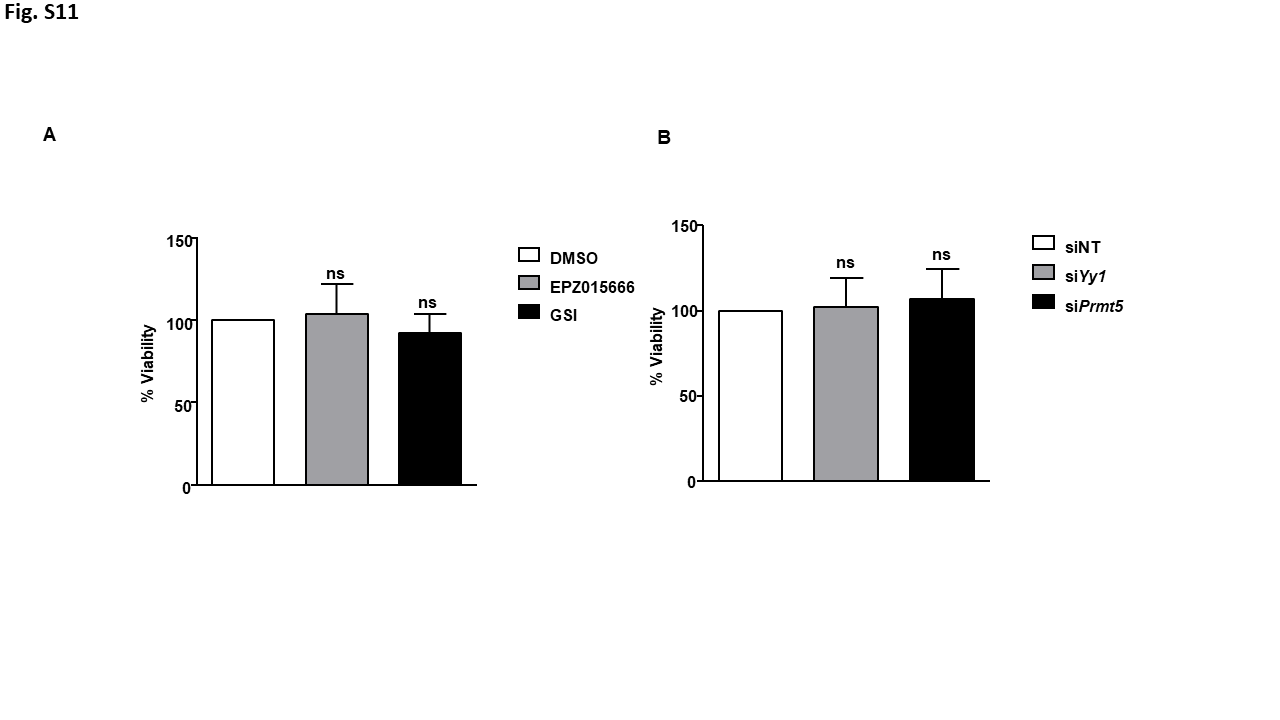

Supplement: S11 Fig — (A) Mouse peritoneal macrophages were treated with PRMT5 inhibitor- EPZ015666 (20 μM) and NOTCH pathway inhibitor- GSI (10μM) for 48 h and percent viability of the cells was determined by MTT assay. (B) Mouse peritoneal macrophages were transfected with NT, Yy1, or Prmt5 siRNAs. Transfected cells were cultured in the medium for 48 h after recovery and subsequently assessed for cell viability by MTT assay. MTT, (3-(4,5-dimethylthiazol-2-yl)-2,5-diphenyltetrazolium bromide); NT, non-targeting; ns, non-significant (Student’s t-test; GraphPad Prism 9.0). (TIF) [file ppat.1010095.s011.TIF]
